# Presynaptic α_2_δ subunits are key organizers of glutamatergic synapses

**DOI:** 10.1073/pnas.1920827118

**Published:** 2021-03-29

**Authors:** Clemens L. Schöpf, Cornelia Ablinger, Stefanie M. Geisler, Ruslan I. Stanika, Marta Campiglio, Walter A. Kaufmann, Benedikt Nimmervoll, Bettina Schlick, Johannes Brockhaus, Markus Missler, Ryuichi Shigemoto, Gerald J. Obermair

**Affiliations:** ^a^Institute of Physiology, Medical University of Innsbruck, A-6020 Innsbruck, Austria;; ^b^Department of Pharmacology and Toxicology, University of Innsbruck, A-6020 Innsbruck, Austria;; ^c^Division of Physiology, Karl Landsteiner University of Health Sciences, A-3500 Krems, Austria;; ^d^Institute of Science and Technology Austria, A-3400 Klosterneuburg, Austria;; ^e^Institute of Anatomy and Molecular Neurobiology, Westfälische Wilhelms University, 48149 Münster, Germany

**Keywords:** synaptic calcium channels, synapse formation, cultured hippocampal neurons, transsynaptic

## Abstract

In nerve cells the genes encoding for α_2_δ subunits of voltage-gated calcium channels have been linked to synaptic functions and neurological disease. Here we show that α_2_δ subunits are essential for the formation and organization of glutamatergic synapses. Using a cellular α_2_δ subunit triple-knockout/knockdown model, we demonstrate a failure in presynaptic differentiation evidenced by defective presynaptic calcium channel clustering and calcium influx, smaller presynaptic active zones, and a strongly reduced accumulation of presynaptic vesicle-associated proteins (synapsin and vGLUT). The presynaptic defect is associated with the downscaling of postsynaptic AMPA receptors and the postsynaptic density. The role of α_2_δ isoforms as synaptic organizers is highly redundant, as each individual α_2_δ isoform can rescue presynaptic calcium channel trafficking and expression of synaptic proteins. Moreover, α_2_δ-2 and α_2_δ-3 with mutated metal ion-dependent adhesion sites can fully rescue presynaptic synapsin expression but only partially calcium channel trafficking, suggesting that the regulatory role of α_2_δ subunits is independent from its role as a calcium channel subunit. Our findings influence the current view on excitatory synapse formation. First, our study suggests that postsynaptic differentiation is secondary to presynaptic differentiation. Second, the dependence of presynaptic differentiation on α_2_δ implicates α_2_δ subunits as potential nucleation points for the organization of synapses. Finally, our results suggest that α_2_δ subunits act as transsynaptic organizers of glutamatergic synapses, thereby aligning the synaptic active zone with the postsynaptic density.

## Significance

Voltage-gated calcium channels are important regulators of neuronal functions, as for example synaptic transmission. Their auxiliary α_2_δ subunits are modulating the calcium currents. Beyond that they have emerged as modulators of synaptic functions. Here, we established a cellular triple knockout/knockdown model in cultured hippocampal neurons by knocking out or knocking down the expression of all three α_2_δ subunits expressed in brain. Our experiments demonstrate that the presynaptic loss of α_2_δ proteins leads to a severe defect in glutamatergic synapse formation, which could be rescued by reintroducing any of the three neuronal α_2_δ isoforms. Thus, our study suggests that α_2_δ proteins are critical regulators of excitatory synapse formation and thereby contributes to the understanding of basic nerve cell functions.

In synapses neurotransmitter release is triggered by the entry of calcium through voltage-gated calcium channels (VGCCs). Neuronal VGCCs consist of an ion-conducting α_1_ subunit and the auxiliary β and α_2_δ subunits. α_2_δ subunits, the targets of the widely prescribed antiepileptic and antiallodynic drugs gabapentin and pregabalin, are membrane-anchored extracellular glycoproteins, which modulate VGCC trafficking and calcium currents (1–5). In nerve cells α_2_δ subunits have been linked to neuropathic pain and epilepsy (4) and they interact with mutant prion proteins (6) and regulate synaptic release probability (7). Importantly, all α_2_δ isoforms are implicated in synaptic functions. Presynaptic effects of α_2_δ-1, for example, may be mediated by an interaction with α-neurexins (8) or *N*-methyl-D-aspartate receptors (e.g., refs. 9 and 10). In contrast, postsynaptic α_2_δ-1 acts as a receptor for thrombospondins (11) and promotes spinogenesis via postsynaptic Rac1 (12). α_2_δ-2 is necessary for normal structure and function of auditory hair cell synapses (13); it has been identified as a regulator of axon growth and hence a suppressor of axonal regeneration (14) and was recently shown to control structure and function of cerebellar climbing fiber synapses (15). A splice variant of α_2_δ-2 regulates postsynaptic GABA_A_ receptor (GABA_A_R) abundance and axonal wiring (16). In invertebrates, α_2_δ loss of function was associated with abnormal presynaptic development in motoneurons (17, 18) and in mice the loss of α_2_δ-3 results in aberrant synapse formation of auditory nerve fibers (19). Finally, α_2_δ-4 is required for the organization of rod and cone photoreceptor synapses (20, 21).

Despite these important functions, knockout mice for α_2_δ-1 and α_2_δ-3 show only mild neurological phenotypes (5, 10, 22–25). In contrast, mutant mice for α_2_δ-2 (ducky) display impaired gait, ataxia, and epileptic seizures (26), all phenotypes consistent with a cerebellar dysfunction due to the predominant expression of α_2_δ-2 in the cerebellum (e.g., ref. 15). Hence, in contrast to the specific functions of α_2_δ isoforms (discussed above) the phenotypes of the available knockout or mutant mouse models suggest a partial functional redundancy in central neurons. Moreover, detailed mechanistic insights into the putative synaptic functions of α_2_δ subunits are complicated by the simultaneous and strong expression of three isoforms (α_2_δ-1 to -3) in neurons of the central nervous system (27).

In this study, by transfecting cultured hippocampal neurons from α_2_δ-2/-3 double-knockout mice with short hairpin RNA (shRNA) against α_2_δ-1, we developed a cellular α_2_δ subunit triple-knockout/knockdown model. Excitatory synapses from these cultures show a severe failure of synaptic vesicle recycling associated with severely reduced presynaptic calcium transients, loss of presynaptic calcium channels and presynaptic vesicle-associated proteins, and a reduced size of the presynaptic active zone (AZ). Lack of presynaptic α_2_δ subunits also induces a failure of postsynaptic PSD-95 and AMPA receptor (AMPAR) localization and a thinning of the postsynaptic density (PSD). Each individual α_2_δ isoform (α_2_δ-1 to -3) could rescue the severe phenotype, revealing the highly redundant role of presynaptic α_2_δ isoforms in glutamatergic synapse formation and differentiation. Together our results show that α_2_δ subunits regulate presynaptic differentiation as well as the transsynaptic alignment of postsynaptic receptors and are thus critical for the function of glutamatergic synapses.

## Results

### Epitope-Tagged α_2_δ Isoforms Localize to Presynaptic Boutons.

Three isoforms of the calcium channel α_2_δ subunit are expressed in hippocampal neurons (27), yet until today it is unclear whether all three isoforms contribute to specific neuronal and synaptic functions. A differential subcellular compartmentalization of α_2_δ isoforms could provide insights into their specific functions. Therefore, we first investigated the localization of hemagglutinin (HA)-epitope-tagged α_2_δ-1, -2, and -3 in cultured hippocampal neurons. To this end a double HA-tag was engineered into N termini of all three α_2_δ subunits cloned from mouse brain complementary DNA (GenBank accession numbers MK327276, MK327277, and MK327280) right after the signal sequence. Live-cell immunolabeling of the HA-epitope allows a direct and, most importantly, comparative analysis of α_2_δ isoform surface expression. Using the same antibody (anti-HA) for quantitatively comparing distinct α_2_δ isoforms provides an important advantage over currently available α_2_δ antibodies, which either do not reliably detect the native proteins (28) or are not suitable for immunocytochemical experiments (29). Although the overall intensity of total surface expression levels differs between isoforms (α_2_δ-2 > α_2_δ-3 > α_2_δ-1), all three isoforms are localized to the somatodendritic and axonal membrane (*SI Appendix*, Fig. S1*A*). In addition, α_2_δ-3 shows a preferential expression in the axon. However, despite these apparent overall differences all α_2_δ isoforms are expressed on the surface of axons and presynaptic membranes (*SI Appendix*, Fig. S1*B*), suggesting that, in principle, all three isoforms can contribute to synaptic functions.

### α_2_δ Subunit Isoforms Are Essential for Survival.

**Fig. 1.**
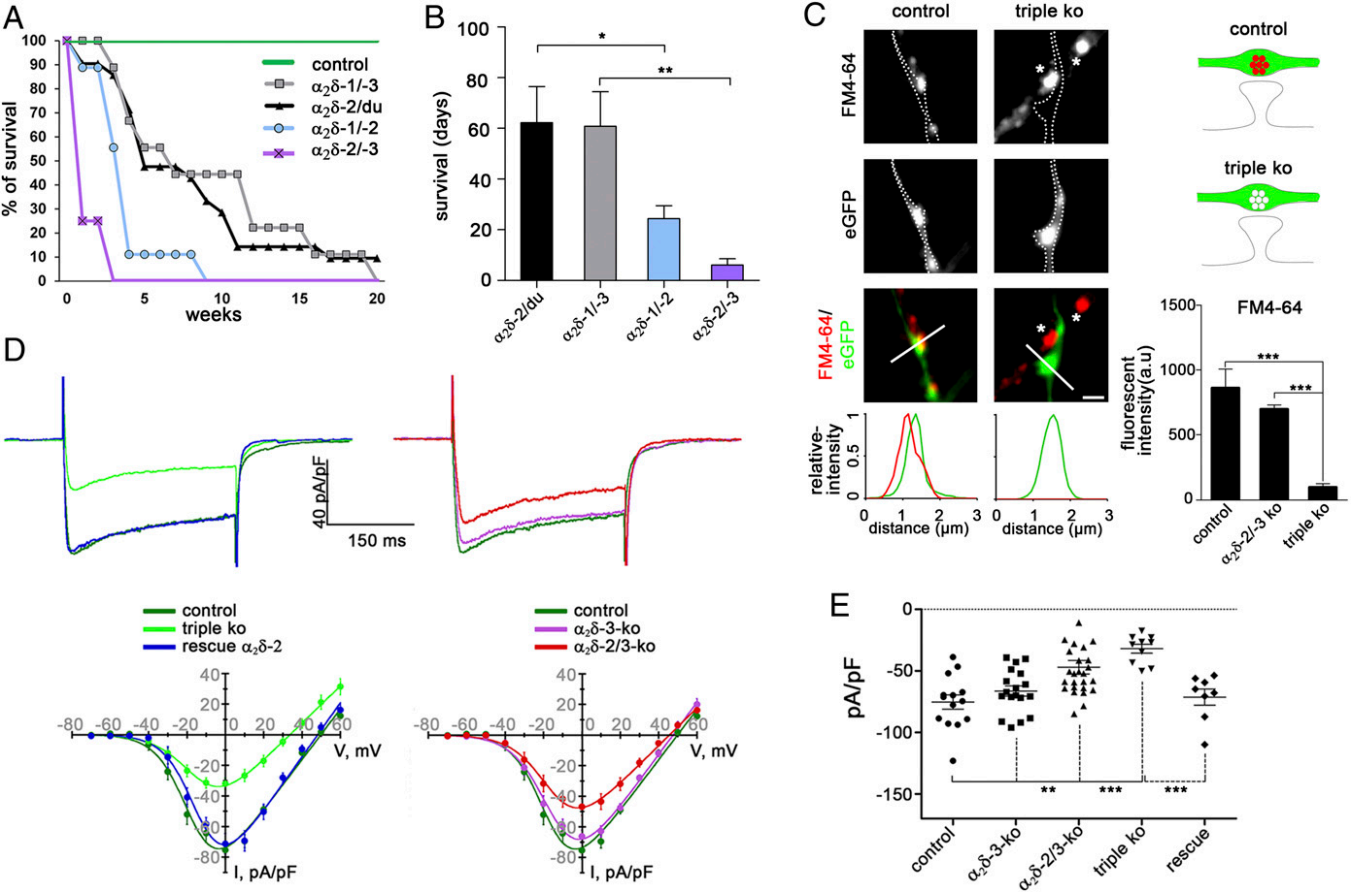
α_2_δ subunits are essential for survival, activity-induced synaptic recycling, and normal calcium current densities. (*A*) The Kaplan–Meier survival curves show an increased mortality in the distinct α_2_δ double-knockout mouse models (*n* = 9 to 21). (*B*) Mean life span was significantly reduced in α_2_δ-1/-2 and α_2_δ-2/-3 double-knockout mice when compared with α_2_δ-1/-3 or ducky mice [ANOVA, *F*_(3,47)_ = 4.7, *P* = 0.006, with Holm–Sidak post hoc test, *P < 0.05, **P < 0.01]. (*C*) Putative synaptic varicosities from α_2_δ TKO/KD neurons failed to load FM4-64 dye upon 60 mM KCl depolarization (outline/triple KO). In contrast, control boutons transfected with eGFP only and nontransfected double-knockout boutons (asterisks) showed robust uptake of the FM-dye. [ANOVA on ranks, H_(2)_= 96.6, *P* < 0.001, with Dunn’s post hoc test, ***P < 0.001, 26 to 110 synapses from two to four culture preparations]. (Scale bar, 1 µm.) (*D*) Current properties of α_2_δ subunit single, double, and TKO/KD cultured hippocampal neurons. Representative Ba^2+^ whole-cell currents at I_max_ (*Upper*) and I/V-curves (*Lower*) recorded from hippocampal neurons. (*Left*) I/V curves reveal a strong reduction of calcium currents in TKO/KD neurons (triple KO), when compared with untransfected wild-type neurons or TKO/KD neurons transfected with α_2_δ-2 (rescue α_2_δ-2). (*Right*) Current densities in α_2_δ-2/-3 double but not in α_2_δ-3 single knockout were also reduced. For I/V curve properties see *SI Appendix*, Table S1. (*E*) Current densities at I_max_ for individual cells [ANOVA, *F*_(4,71)_ = 11.3, *P* < 0.001, with Holm–Sidak post hoc test, **P < 0.01, ***P < 0.001, 8 to 26 cells from five culture preparations]. Horizontal lines represent means and error bars SEM.

With the exception of the α_2_δ-2 mutant mouse ducky, knockout mice for α_2_δ-1 and α_2_δ-3 display only mild neuronal phenotypes, suggesting a potential and at least partial functional redundancy (discussed above). Therefore, in order to gain insight into the functional diversity of α_2_δ subunits, we generated double-knockout mice by pairwise cross-breeding single-knockout (α_2_δ-1 and α_2_δ-3) and mutant (α_2_δ-2^du^) mice (29). While α_2_δ-1/-3 knockout mice are viable for up to 3 mo, similar to ducky mice, α_2_δ-1/-2 and α_2_δ-2/-3 knockout mice have a strongly reduced lifespan (Fig. 1 *A* and *B*). A significant proportion of these mice require application of humane endpoints within the first postnatal week, mainly due to malnutrition associated with a poor general condition. Together this shows that α_2_δ subunits serve essential functions and are necessary for survival. Moreover, the increased severity of the phenotype in double- compared with single-knockout mice also supports the idea that α_2_δ subunits act in part redundantly.

### Establishing a Cellular α_2_δ-Subunit Triple-Knockout/Knockdown Model.

In order to study a potential functional redundancy of α_2_δ subunits we next developed a cellular α_2_δ triple-knockout/knockdown model system by transfecting cultured hippocampal neurons from α_2_δ-2/-3 double-knockout mice with shRNA against α_2_δ-1. To this end we first confirmed efficient shRNA knockdown of α_2_δ-1 in two independent experimental settings. First, shRNA against α_2_δ-1 (30, 31) significantly reduced the surface expression of a heterologously expressed α_2_δ-1 isoform bearing an extracellular phluorin-tag (superecliptic phluorin, SEP; *SI Appendix*, Fig. S2 *A* and *B*); however, due to the experimental overexpression low levels of this α_2_δ-1 isoform were still detectable (37% of control; *SI Appendix*, Fig. S2 *A* and *B*). Second, qPCR analysis of cultured hippocampal neurons virally infected with α_2_δ-1 shRNA revealed an overall 80% knockdown of α_2_δ-1 messenger RNA (mRNA) compared with untransfected (wild-type) neurons or neurons expressing scrambled control shRNA (*SI Appendix*, Fig. S2*C*). Considering a ∼90% infection efficiency, confirmed by enhanced green fluorescent protein (eGFP) expression from the same viral vector, shRNA robustly knocked down mRNA in the vast majority of infected neurons. Most importantly, shRNA knockdown of α_2_δ-1 did not affect the expression levels of the other α_2_δ isoforms (*SI Appendix*, Fig. S2*C*). In order to evaluate potential compensatory mechanisms, we also quantified mRNA levels of all α_2_δ isoforms in hippocampal tissue from 8-wk-old single-knockout mice. Similar to α_2_δ-1 knockdown, neither loss of α_2_δ-2 nor of α_2_δ-3 induced compensational changes in the expression levels of the other isoforms (*SI Appendix*, Fig. S2 *D* and *E*).

α_2_δ-2/-3 double-knockout mice were generated by cross-breeding double heterozygous α_2_δ-2^+/du^/α_2_δ-3^+/−^ mice (*SI Appendix*, Fig. S3*A*). The predicted Mendelian ratio for double-knockout mice is 6.25%, however, the experimentally determined ratio was only ∼3% (see legend of *SI Appendix*, Fig. S3). Neonatal pups (postnatal day [P] 0 to 2) were individually marked by paw tattooing and genotyped for the α_2_δ-2 and α_2_δ-3 alleles (*SI Appendix*, Fig. S3 *B* and *C*). Due to the large genomic rearrangement in ducky mice, genotyping of the ducky mutation required a confirmation employing a copy-number-counting qPCR approach (*SI Appendix*, Fig. S3*D*). Ultimately, α_2_δ triple loss-of-function hippocampal neurons were established by transfecting confirmed α_2_δ-2/-3 double-knockout cultures with α_2_δ-1 shRNA and eGFP (*SI Appendix*, Fig. S3*E*).

### Failure of Presynaptic Differentiation in α_2_δ Subunit Triple-Knockout/Knockdown Neurons.

In cultured hippocampal neurons from α_2_δ-2/-3 double-knockout mice, shRNA-transfected neurons (α_2_δ-2/-3 double-knockout with α_2_δ-1 shRNA knockdown, further referred to as α_2_δ TKO/KD) can be easily identified by the expression of soluble eGFP. Most importantly, in this experimental setting isolated axons and synaptic varicosities from transfected α_2_δ TKO/KD neurons can be directly compared with untransfected neighboring neurons, which still express α_2_δ-1 (*SI Appendix*, Fig. S4). Axons from cultured hippocampal neurons display axonal varicosities which can be morphologically identified by the eGFP fluorescence. Such varicosities are typically representing presynaptic boutons, as confirmed by the clustering of presynaptic proteins (e.g., Ca_V_2.1 channels and synapsin; *SI Appendix*, Fig. S4, *Left*). Axons from α_2_δ TKO/KD neurons display similar axonal varicosities, mostly found located along dendritic processes of nontransfected neighboring double-knockout cells (*SI Appendix*, Fig. S4, *Right*). In order to test whether these boutons represent functional synapses capable of vesicle recycling we quantified the extent of depolarization-induced uptake of the styryl membrane dye FM4-64. Upon a 60 mM [K^+^]-induced depolarization 68% of the axonal varicosities of α_2_δ TKO/KD neurons completely failed to take up FM dye and loading of the remaining 32% was strongly decreased (Fig. 1*C*). In contrast, neighboring untransfected (α_2_δ-1–containing) synapses (Fig. 1 *C*, *Right*, asterisks) and eGFP-transfected control neurons were readily stained with FM4-64 upon high [K^+^] treatment. This apparent failure of synaptic vesicle recycling pointed toward a severe defect in presynaptic calcium channel functions.

Indeed, voltage-clamp analysis of total somatic calcium currents identified a marked reduction of current densities by 58% (Fig. 1 *D* and *E*) and of the maximal conductance by 37% (*SI Appendix*, Fig. S5) but no change in the voltage-dependence of activation and half-maximal activation of α_2_δ TKO/KD compared with α_2_δ-3 single-knockout or wild-type control neurons (Fig. 1 *D* and *E* and *SI Appendix*, Table S1 and Fig. S5). Notably, current densities and maximal conductance were also reduced in α_2_δ-2/-3 double-knockout neurons (by 32% and 23%, respectively; Fig. 1*E* and *SI Appendix*, Fig. S5*C*), however without a concomitant failure in FM dye uptake (Fig. 1*C*). The homologous reconstitution of α_2_δ-2 in TKO/KD neurons fully rescued the currents back to wild-type levels (Fig. 1 *D* and *E* and *SI Appendix*, Table S1), while the sole presence of α_2_δ-1 in the α_2_δ-2/-3 double-knockout condition could not fully compensate the effects on total somatodendritic currents (Fig. 1*D*, red trace). Importantly, to avoid any possible influence of the HA-epitope tag (discussed above) on α_2_δ isoform function, all rescue experiments were performed with wild-type, untagged α_2_δ subunits.

**Fig. 2.**
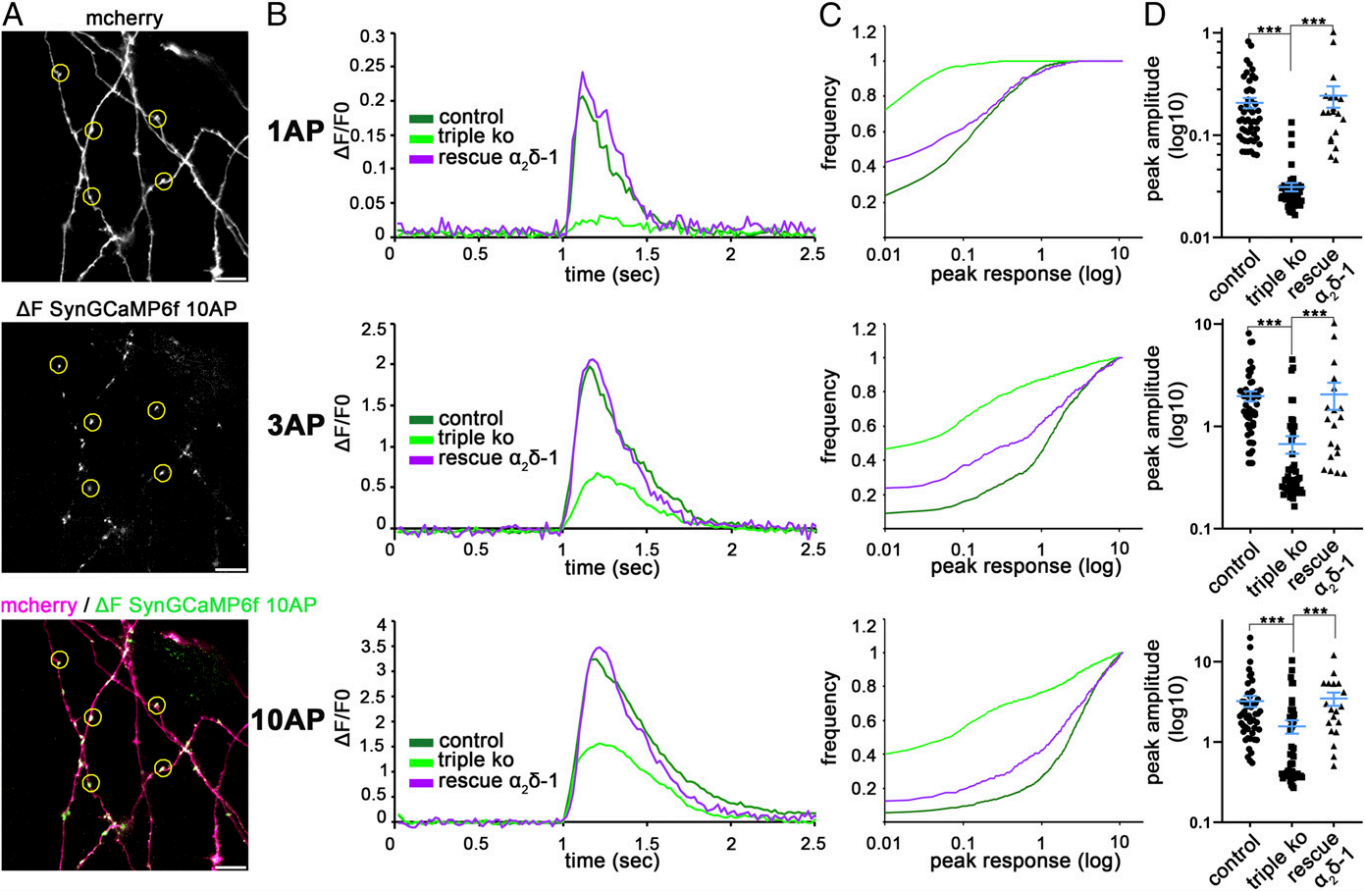
α_2_δ subunits are essential for activity-induced presynaptic calcium transients. (*A*) Putative synaptic varicosities from neurons cotransfected with SynGCaMP6f and mCherry were selected in Fiji/ImageJ using the ROI tool (yellow circles). For quantification of the presynaptic calcium transients, regions were transferred to the corresponding recordings of SynGCaMP6f fluorescence, shown here as fluorescence change by subtracting an averaged control image from an image averaged around the maximal response. (Scale bar, 10 µm.) (*B*) SynGCaMP6f fluorescence (ΔF/F0) for stimulations with 1 AP, 3 APs, and 10 APs at 50 Hz. Lines show the mean fluorescence traces for control (50 cells from three independent cultures), TKO/KD (triple KO, 50 cells from three independent cultures), and the rescue condition expressing α_2_δ-1 together with SynGCaMP6f and mCherry (19 cells from two independent cultures). (*C*) Cumulative frequency distribution histograms of peak fluorescent responses (ΔF/F0) from all recorded putative synaptic varicosities of α_2_δ TKO/KD (light green), double-heterozygous control (dark green), and α_2_δ-1 overexpressing TKO/KD neurons (rescue α_2_δ-1, purple) in response to stimulations with 1, 3, or 10 APs (number of synapses: control, 1,100; triple KO, 1,100; rescue α_2_δ-1, 418). (*D*) Quantification of peak fluorescent amplitudes in response to stimulations with 1, 3, or 10 APs. Each dot represents the mean of 22 synapses from one neuron [Kruskal–Wallis ANOVA with Dunn’s multiple comparison test: 1 AP: H_(3,__119)_ = 81, *P* < 0.0001; 3 AP: H_(3,__119)_ = 48, *P* < 0.0001; 10 AP: H_(3,__119)_ = 26, *P* < 0.0001; post hoc test: ***P ≤ 0.001; 50 (control, triple KO) and 19 (rescue α_2_δ-1) cells from three and two independent culture preparations].

While reduced somatic calcium channel activity was to be expected in an α_2_δ-null model, the complete failure of FM dye uptake suggests a more severe failure of synaptic vesicle recycling. Therefore, we next tested the consequences of α_2_δ TKO/KD on presynaptic calcium signals. To this end neurons were transfected with the genetically encoded calcium indicator GCaMP6f, coupled to synaptophysin and expressed under the control of a synapsin promoter (32), together with soluble mCherry to outline neuronal and axonal morphology (Fig. 2*A*). Presynaptic calcium signals (ΔF/F0) were recorded and analyzed in putative presynaptic boutons (axonal varicosities, discussed above) in response to field stimulation triggering 1, 3, or 10 action potentials (APs) at a frequency of 50 Hz (Fig. 2*B* and Movies S1 and S2). α_2_δ TKO/KD resulted in a reduction of mean peak amplitudes to 15%, 34%, and 48% in response to 1, 3, and 10 APs, respectively, in comparison with double-heterozygous control neurons (Fig. 2 *B* and *D*). Ectopic overexpression of α_2_δ-1, which was reduced but not eliminated by shRNA (*SI Appendix*, Fig. S2*B*), rescued mean peak amplitudes to 117%, 104%, and 107% of control. Even more strikingly, frequency distribution histograms of peak responses of all synapses (Fig. 2*C*) show that 73%, 44%, and 39% of TKO/KD boutons failed to show any calcium transient in response to 1, 3, and 10 APs. In contrast, failures of heterozygous control neurons were observed in only 24%, 8%, and 5%, and those of α_2_δ-1-rescued neurons in only 43%, 23%, and 12% of synapses, each in response to 1, 3, and 10 APs, respectively. Together these results demonstrate a severe reduction of presynaptic calcium influx and hence suggest a defect in presynaptic calcium channel clustering in α_2_δ TKO/KD boutons.

**Fig. 3.**
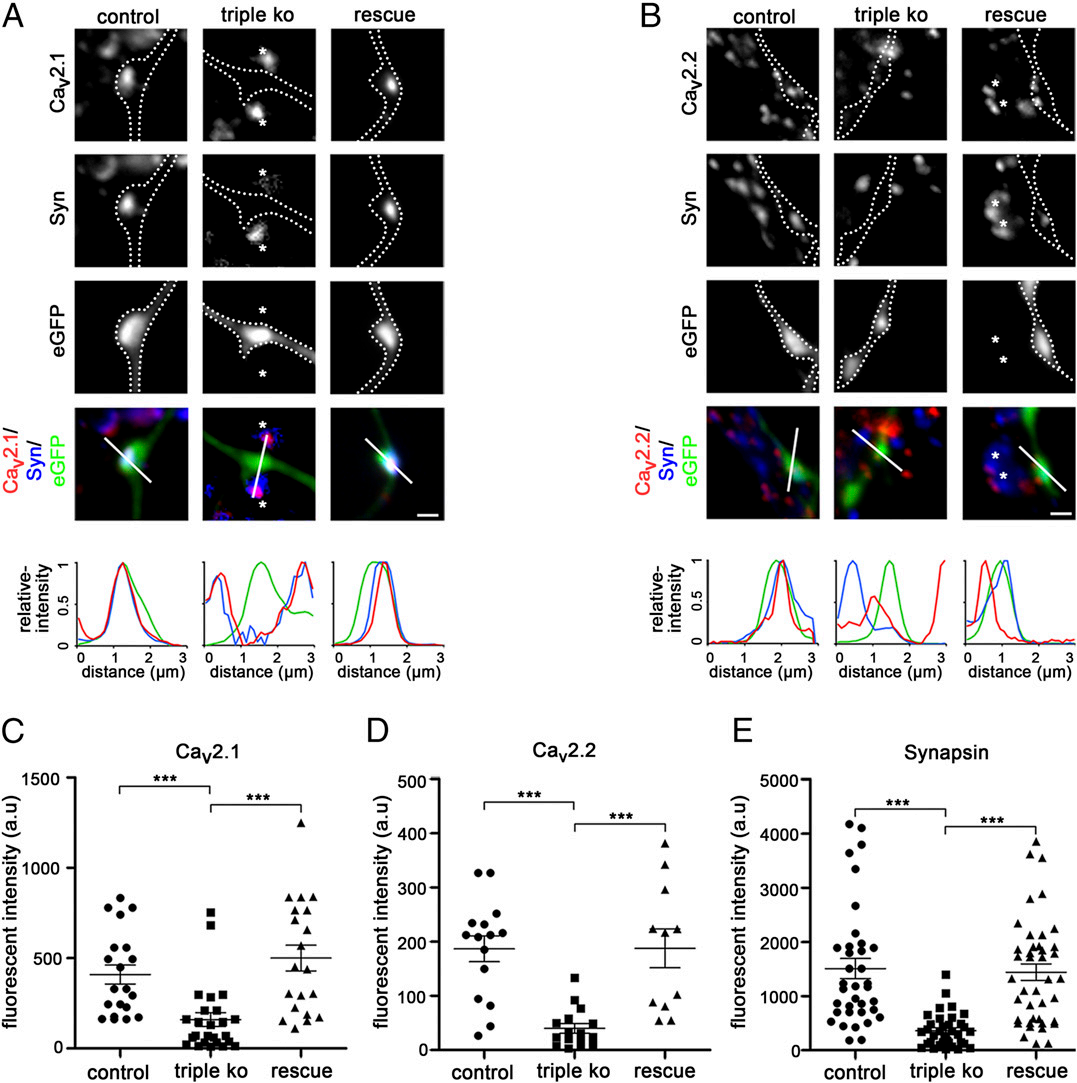
Failure of presynaptic calcium channel clustering and synapsin accumulation in α_2_δ subunit triple-knockout/knockdown neurons. (*A* and *B*) Immunofluoresence analysis of axonal varicosities from wild-type neurons (control, neurons transfected with eGFP only), TKO/KD neurons (triple KO, α_2_δ-2/-3 double-knockout neurons transfected with shRNA-α_2_δ-1 plus eGFP), and TKO/KD neurons expressing α_2_δ-2 (rescue, α_2_δ-2/-3 double-knockout neurons transfected with shRNA-α_2_δ-1 plus eGFP and α_2_δ-2). Putative presynaptic boutons were identified as eGFP-filled axonal varicosities along dendrites of untransfected neurons (*SI Appendix*, Fig. S4) and outlined with a dashed line. Immunolabeling revealed a failure in the clustering of presynaptic P/Q- (*A*, Ca_V_2.1) and N-type (*B*, Ca_V_2.2) channels as well as in the accumulation of presynaptic synapsin in varicosities from α_2_δ TKO/KD neurons (*Middle*). In contrast, wild-type control neurons (*Left*) displayed a clear colocalization of the calcium channel clusters with synapsin in the eGFP-filled boutons. The linescan patterns recorded along the indicated line support these observations. Note that the sole expression of α_2_δ-2 (*Right*) or the sole presence of α_2_δ-1 in synapses from neighboring α_2_δ-2/-3 double-knockout neurons (asterisks in *A* and *B*) suffices to fully rescue presynaptic calcium channel clustering and synapsin accumulation. (*C*–*E*) Quantification of the fluorescence intensities of presynaptic Ca_V_2.1 (*C*), Ca_V_2.2 (*D*), and synapsin (*E*) clustering in control, TKO/KD, and α_2_δ-2–expressing (rescue) TKO/KD neurons [ANOVA with Holm–Sidak post hoc test, ***P < 0.001; Ca_V_2.1: *F*_(2,__58)_ = 10.8, *P* < 0.001, 16 to 25 cells from four to six culture preparations; Ca_V_2.2: *F*_(2,__37)_ = 13.7, *P* < 0.001, 11 to 16, two to four; synapsin: *F*_(2,__99)_ = 15.5, *P* < 0.001, 30 to 36, five to eight; horizontal lines represent means and error bars SEM]. (Scale bars, 1 µm.)

Therefore, we next employed immunocytochemistry to test whether and to what extent the synaptic localization of presynaptic P/Q- (Ca_V_2.1) and N-type (Ca_V_2.2) calcium channels was affected (Fig. 3 *A* and *B*). Strikingly, 61% and 40% of the axonal TKO/KD varicosities lacked detectable staining for Ca_V_2.1 and Ca_V_2.2, respectively. The remaining axonal boutons showed a strong and significant reduction of presynaptic labeling intensities (Fig. 3 *C* and *D*). In agreement with defective synaptic vesicle recycling, these boutons were also deficient in synapsin staining (complete loss in 45% of the analyzed boutons; Fig. 3*E*). The strongly reduced presynaptic calcium channel abundance in α_2_δ TKO/KD varicosities is in line with the major role of α_2_δ subunits in enhancing calcium channel trafficking (33). However, the surprising loss of synapsin staining suggests that the lack of α_2_δ subunits also grossly affects presynaptic differentiation.

### Presynaptic α_2_δ Subunits Regulate Pre- and Postsynaptic Differentiation of Excitatory Glutamatergic Synapses.

**Fig. 4.**
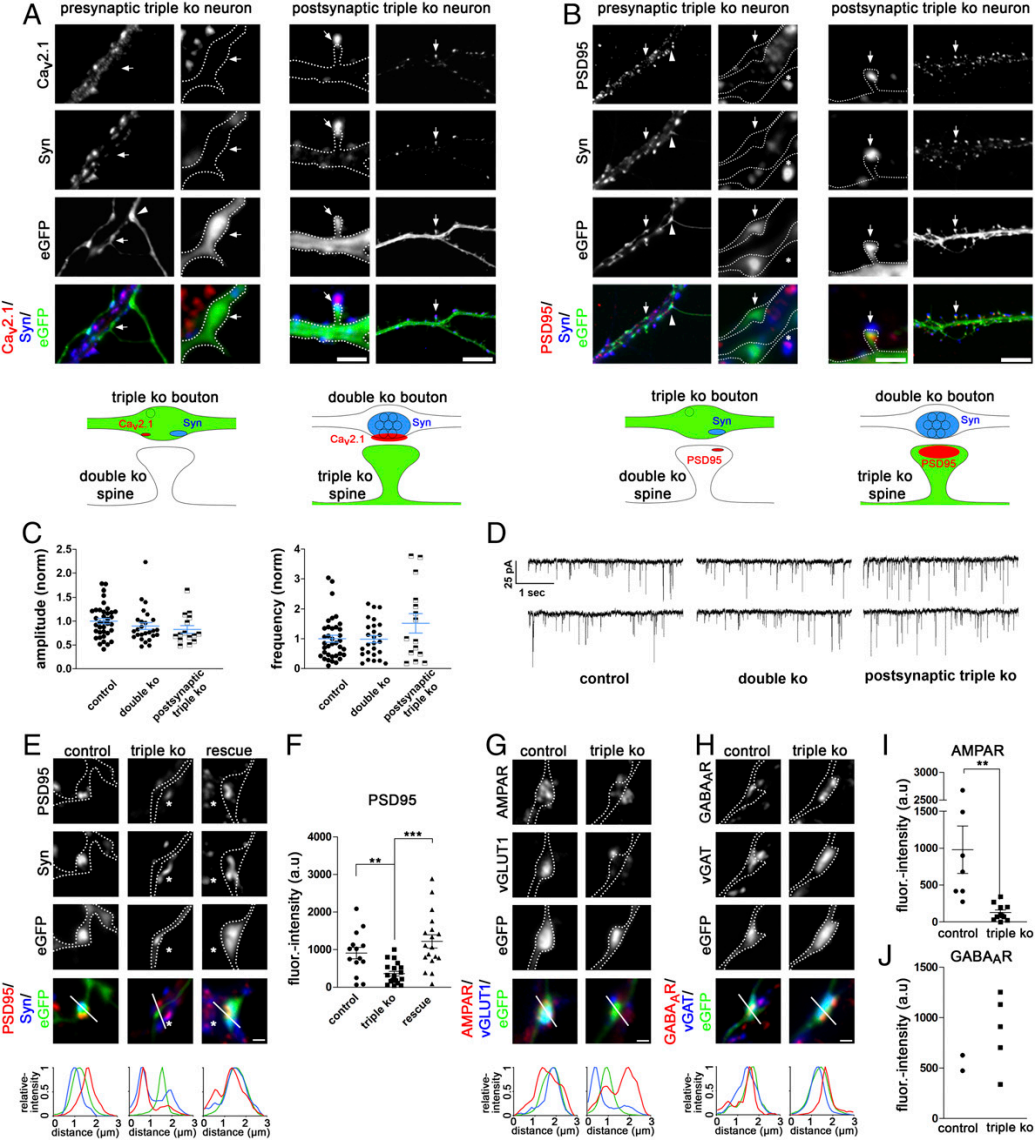
Presynaptic α_2_δ subunits mediate glutamatergic synapse formation and transsynaptic differentiation. (*A* and *B*) Immunofluoresence micrographs of axonal varicosities from presynaptic α_2_δ TKO/KD neurons (eGFP-positive axonal varicosities, *Left*, arrows) as well as dendrites from postsynaptic TKO/KD neurons (eGFP-positive dendrites, *Right*, arrows). Axonal varicosities and dendrites are outlined by a dashed line and arrowheads mark exemplary innervating triple-knockout axons. The sketches summarize the observed labeling patterns. (*A*) α_2_δ TKO/KD neurons display a failure of presynaptic Ca_v_2.1 channel and synapsin clustering exclusively in presynaptic axonal varicosities (arrows and sketch, *Left*). In contrast, postsynaptic TKO/KD neurons developed dendritic spines opposite presynaptic boutons containing Ca_v_2.1 and synapsin clusters (arrows and sketch, *Right*) formed by axons from α_2_δ-2/-3 double-knockout neurons still containing α_2_δ-1. (*B*) Presynaptic α_2_δ TKO/KD induces a failure of the postsynaptic PSD-95 clustering indicating a transsynaptic action of α_2_δ subunits (arrows and sketch, *Left*). Conversely, postsynaptic TKO/KD neurons still receive proper synaptic input from neighboring α_2_δ-1 containing neurons as indicated by presynaptic synapsin and postsynaptic PSD-95 colocalized on TKO/KD dendritic spines (arrows and sketch, *Right*). (Scale bars, 2 µm and 8 µm.) (*C* and *D*) mEPSC recordings and analysis from control (double heterozygous), double-knockout (double KO, α_2_δ-2/-3 double-knockout), and postsynaptic TKO/KD neurons (triple KO) receiving synaptic input from neighboring double-knockout neurons (see *A*, *Right*). (*C*) Quantification of mEPSC amplitues (*Left*) and frequencies (*Right*). Amplitudes and frequencies of mEPSCs for each condition were normalized to the mean value of control condition for each individual experiment. [Amplitude: one-way ANOVA, *F*_(2,75)_ = 1.56, *P* = 0.22; frequency: ANOVA, *F*_(2,75)_ = 2.48, *P* = 0.09; *n* = 37 (control]), 26 (double KO), and 15 (postsynaptic triple KO) from four, four, and two culture preparations, respectively]. (*D*) Representative traces of mEPSC for condition described in *C*. (*E* and *F*) Failure of postsynaptic PSD-95 labeling opposite α_2_δ TKO/KD boutons. Similar to the presynaptic proteins (see Fig. 3) the sole expression of α_2_δ-2 (rescue, right column) or the sole presence of α_2_δ-1 in synapses from neighboring α_2_δ-2/-3 double-knockout neurons (asterisks in *E*, middle column, linescans) fully rescued postsynaptic PSD-95 clustering [ANOVA, *F*_(2,__49)_ = 11.7, *P* < 0.001, with Holm–Sidak post hoc test, **P < 0.01, ***P < 0.001; 14 to 20 cells from three to four culture preparations). (*G* and *I*) The defect in synaptogenesis caused by loss of α_2_δ subunits specifically affects glutamatergic synapses, indicated by reduced fluorescent intensity of vGLUT1/AMPAR labeling (outline/linescan; *t* test, *t*_(15)_ = 3.1, **P < 0.01; 7 and 10 cells from two and three culture preparations). (*H* and *J*) In contrast, vGAT/GABA_A_R labeling in GABAergic synapses did not seem to be reduced in α_2_δ TKO/KD neurons (outline/linescan; two and five cells from one and two culture preparations). Error bars indicate SEM. (Scale bars, 1 µm.)

By acting as a thrombospondin receptor, α_2_δ-1 has previously been suggested to contribute to synaptogenesis by a postsynaptic mechanism (11, 12). Therefore, in order to distinguish between the proposed postsynaptic mechanism and the defect in presynaptic differentiation observed here, we examined α_2_δ TKO/KD neurons connected to neighboring nontransfected double-knockout neurons still expressing α_2_δ-1 (*SI Appendix*, Fig. S4). In this experimental paradigm, eGFP-positive axonal processes of presynaptic TKO/KD neurons (Fig. 4 *A* and *B*, *Left* and sketches) can be clearly distinguished from eGFP-positive dendrites of postsynaptic TKO/KD neurons (Fig. 4 *A* and *B*, *Right* and sketches). These experiments demonstrate that synapse differentiation fails when the presynaptic neuron lacks all α_2_δ subunits (Fig. 4 *A* and *B*, *Left*). On the other hand, postsynaptic α_2_δ TKO/KD neurons can still form dendritic spines and receive synaptic inputs from neighboring double-knockout neurons expressing α_2_δ-1 (Fig. 4 *A* and *B*, *Right*). This observation is confirmed by recording miniature excitatory postsynaptic currents (mEPSC) from postsynaptic α_2_δ TKO/KD neurons in comparison with heterozygous control or double-knockout neurons (Fig. 4 *C* and *D*). Neither mEPSC frequency nor amplitude is reduced in putative TKO/KD synapses, strengthening the conclusion that postsynaptic α_2_δ TKO/KD neurons can still receive synaptic inputs from neighboring double-knockout neurons.

**Fig. 5.**
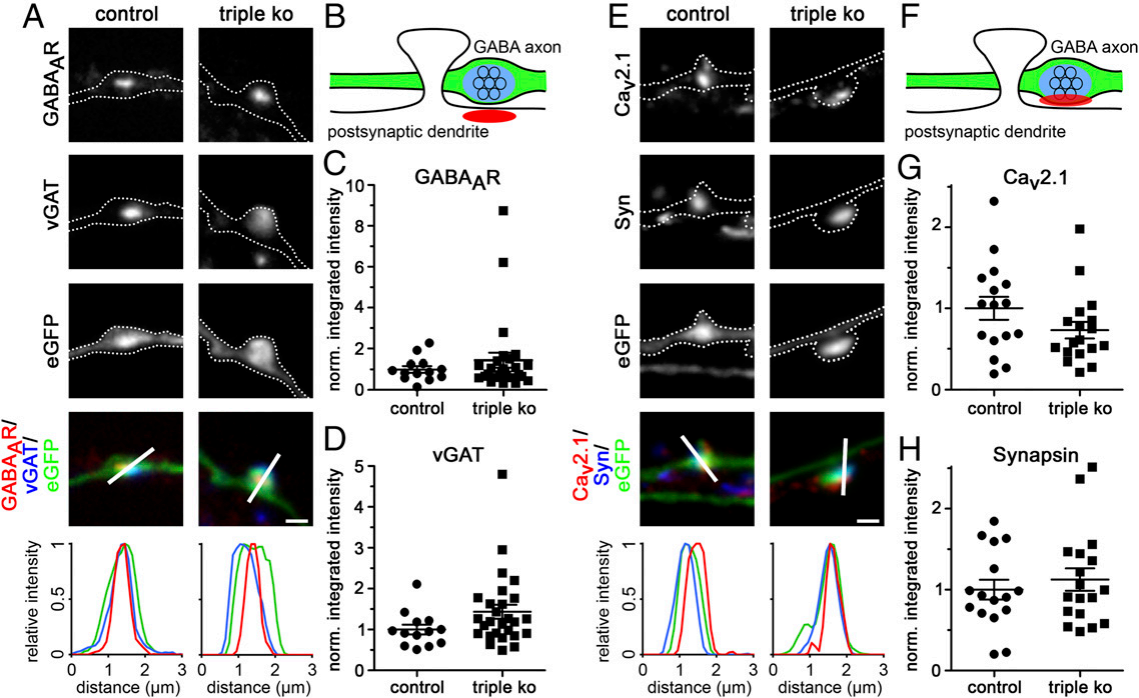
Presynaptic α_2_δ subunit triple-knockout/knockdown does not affect pre- and postsynaptic differentiation in GABAergic synapses. (*A* and *E*) Representative immunofluoresence micrographs of axonal varicosities from presynaptic α_2_δ-3 knockout (control) or TKO/KD (triple KO) cultured GABAergic MSNs. Transfected neurons (22 to 24 DIV) were immunolabeled for vGAT and the GABA_A_R (*A*) and Ca_V_2.1 and synapsin (*E*). Colocalization of fluorescence signals within eGFP-filled axonal varicosities (axons are outlined with dashed lines) was analyzed using line scans. (*B* and *F*) Sketches depicting the expected staining patterns in *A* and *E*, respectively. (*C*, *D*, *G*, and *H*) Quantification of the respective fluorescence intensities in control and TKO/KD neurons (*t* test, GABA_A_R: *t*_(38)_ = 0.8, *P* = 0.41, 13 to 27 cells from three culture preparations; vGAT: *t*_(38)_ = 1.7, *P* = 0.10, 13 to 27 cells from three culture preparations; Ca_V_2.1: *t*_(32)_ = 1.6, *P* = 0.13, 16 to 18 cells from two culture preparations; synapsin: *t*_(32)_ = 0.7, *P* = 0.51, 16 to 18 cells from two culture preparations). Values for individual cells (dots) and means (lines) ± SEM are shown. Values were normalized to control (α_2_δ-3 knockout) within each culture preparation. (Scale bars, 1 µm.)

The presynaptic defect in synapse formation also induced a failure in the postsynaptic differentiation: Boutons devoid of calcium channels or synapsin were either not juxtaposed to PSD-95 clusters at all (Fig. 4*E*) or the PSD-95 labeling was strongly reduced (Fig. 4*F*). Similar to the marked reduction of presynaptic synapsin and calcium channel labeling, PSD-95 was completely absent in 58% of the analyzed α_2_δ TKO/KD synapses. Thus, in addition to the failure in presynaptic differentiation, the lack of presynaptic α_2_δ subunits also induced a failure in postsynaptic differentiation. For analyzing whether presynaptic α_2_δ subunits are required for both excitatory and inhibitory synapse formation, we immunolabeled α_2_δ TKO/KD and control neurons for respective components of the presynaptic vesicle compartment and postsynaptic receptors (Fig. 4 *G* and *H*). In excitatory glutamatergic neurons the lack of presynaptic staining for the vesicular glutamate transporter type 1 (vGLUT1; for quantification see *SI Appendix*, Fig. S6) goes along with strongly reduced clustering of postsynaptic AMPARs in TKO/KD synapses (Fig. 4 *G* and *I*). Conversely, α_2_δ TKO/KD synapses from GABAergic neurons still seem to express the presynaptic vesicular GABA transporter (vGAT) and display postsynaptic clustering of GABA_A_R (Fig. 4 *H* and *J*). However, it is important to note that due to the low abundance of GABAergic neurons (∼5 to 10% of all cultured hippocampal neurons), the extremely low availability of α_2_δ-2/-3 double-knockout offspring (on average only two to five culture preparations are possible per year), and the necessity of shRNA transfection, we could only analyze two cells for control and five cells for TKO/KD conditions. Therefore, to confirm this finding we also analyzed the abundance of pre- and postsynaptic proteins in control (α_2_δ-3 knockout) and α_2_δ TKO/KD cultured GABAergic striatal medium spiny neurons (Fig. 5) (16). In contrast to glutamatergic hippocampal neurons, α_2_δ TKO/KD in GABAergic medium spiny neurons does not affect the abundance of presynaptic vGAT (Fig. 5*D*) and synapsin (Fig. 5*H*), the presynaptic bouton size (*SI Appendix*, Fig. S7), or postsynaptic GABA_A_-receptor clustering (Fig. 5*C* and *SI Appendix*, Fig. S7). However, although not statistically significant, there was a tendency for reduced presynaptic Ca_V_2.1 labeling (Fig. 5*G*). Together this demonstrates that the severe consequence of presynaptic α_2_δ TKO/KD is specific to excitatory glutamatergic neurons.

### α_2_δ Subunit Triple Knockout/Knockdown Affects the Pre- and Postsynaptic Ultrastructure.

**Fig. 6.**
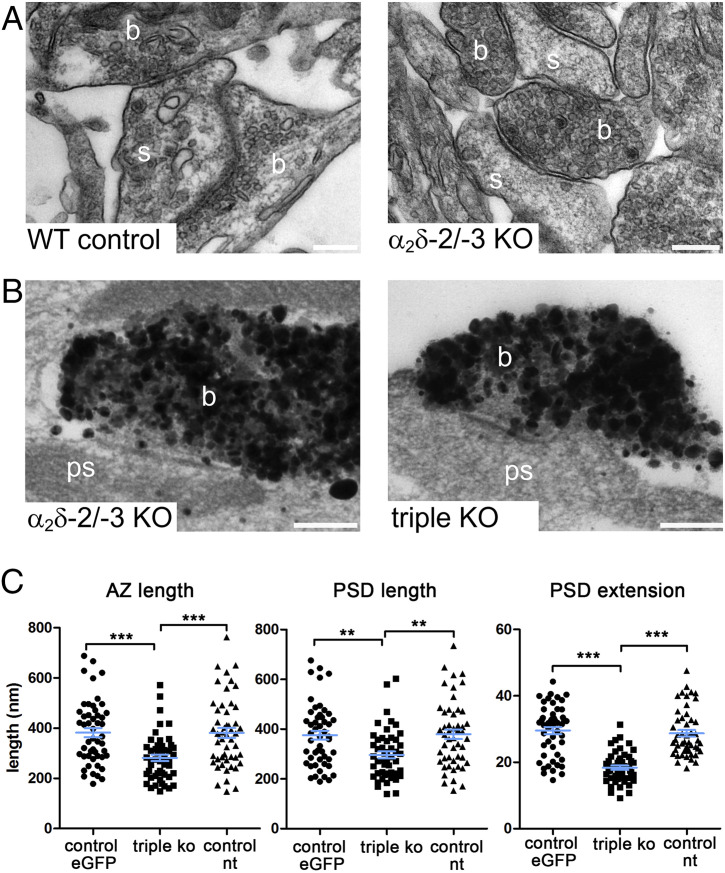
Ultrastructural analysis of pre- and postsynaptic specializations in excitatory α_2_δ subunit triple-knockout/knockdown synapses. (*A*) Exemplary EM micrographs of synaptic structures show similar presynaptic and postsynaptic differentiation in wild-type control (*Left*) and α_2_δ-2/-3 double-knockout (α_2_δ-2/-3 KO) cultured hippocampal neurons (for statistics see text). (*B*) Exemplary EM micrographs from silver-amplified eGFP-immunogold-stained presynaptic boutons and the corresponding postsynaptic region from double (α_2_δ-2/-3) and TKO/KD (triple KO) synapses. (*C*) Quantitative analyses showing that both the length of the AZ (*Left*) and the PSD (*Middle*) were significantly reduced in TKO/KD compared with eGFP-transfected α_2_δ-2/-3 double-knockout synapses in separate culture preparations (control eGFP) and nontransfected neighboring synapses within the same coverglass (control nt). In addition to the AZ and PSD length also the thickness, particularly the extension of the PSD from the membrane into the cytosol, was strongly reduced in TKO/KD compared with the respective control synapses [ANOVA with Tukey post hoc test, **P < 0.01, ***P < 0.001; AZ length: *F*_(2,147)_ = 11.3, *P* < 0.001; PSD length: *F*_(2,147)_ = 7.5, *P* < 0.001; PSD extension: *F*_(2,147)_ = 44.6, *P* < 0.001. Horizontal lines represent means and error bars SEM]. Abbreviations in EM micrographs: b, presynaptic bouton; s, dendritic spine; ps, postsynaptic compartment. (Scale bars, 200 nm.)

Immunofluorescence labeling identified a strong reduction in the abundance of presynaptic and postsynaptic proteins in glutamatergic synapses of α_2_δ TKO/KD neurons. In order to test whether these presynaptic effects are associated with ultrastructural alterations we performed classical transmission electron microscopy (TEM) and preembedding immunoelectron microscopy. Classical TEM analysis revealed the necessity for immunolabeling shRNA-α_2_δ-1/eGFP transfected double-knockout neurons in order to reliably identify the sparsely distributed α_2_δ TKO/KD synapses weak in morphological cues. The strong immunolabeling for eGFP with the contrast intense silver-amplified gold particles, however, obscured the presynaptic ultrastructure and rendered reliable analysis of synaptic vesicle content and localization impossible. For quantifying size and extension of synaptic specializations, we first compared synapses of nonlabeled wild-type control and α_2_δ-2/-3 double-knockout neurons (Fig. 6*A*). Analyses of 40 synapses in each condition revealed that the length of the AZ and the PSD, the AZ/PSD ratio, as well as the PSD thickness (extension from the membrane into the cytosol) were indistinguishable between control and double-knockout neurons (mean ± SEM in nanometers, unpaired *t* test; AZ: control, 433 ± 22, double-KO, 433 ± 20, *P* = 0.99; PSD: control, 440 ± 23, double-KO, 436 ± 20, *P* = 0.89; PSD extension: control, 28.5 ± 1.4; double-KO, 27.2 ± 1.1, *P* = 0.49; AZ/PSD ratio: control, 0.986 ± 0.004, double-KO, 0.995 ± 0.006; *P* = 0.19). We next performed the same analysis on eGFP-immunostained double-knockout (control eGFP) and TKO/KD (triple KO) synapses (Fig. 6*B*). As an additional control, we measured the respective AZ and PSD parameters of nontransfected neighboring synapses (control nt), which are all double-knockout for α_2_δ-2/-3. Both AZ and PSD lengths were significantly reduced by ∼25% in α_2_δ TKO/KD synapses (Fig. 6 *C*, *Left* and *Middle*); however, the AZ/PSD ratio was not altered [AZ/PSD ratio: control eGFP, 1.00 ± 0.01, triple KO, 1.05 ± 0.04; control nt, 0.98 ± 0.04; ANOVA, *F*_(2,__147)_ = 1.45, *P* = 0.24]. This suggests that reductions in the presynaptic AZ caused by lack of α_2_δ subunits are directly affecting the size of the PSD. Control measurements in nontransfected synapses (control nt) were indistinguishable from eGFP-transfected α_2_δ-2/-3 double-knockout neurons (control eGFP). The extension of the PSD from the synaptic membrane into the cytosol was reduced by 40% in TKO/KD synapses compared with both controls (Fig. 6 *C*, *Right*). Taken together, these measurements reveal that presynaptic α_2_δ TKO/KD reduces the sizes of the presynaptic AZ and PSD as well as the thickness of the PSD (Fig. 4*E*).

### The α_2_δ Subunit Triple-Knockout/Knockdown Phenotype Can Be Rescued by Overexpression of α_2_δ-1, -2, and -3.

**Fig. 7.**
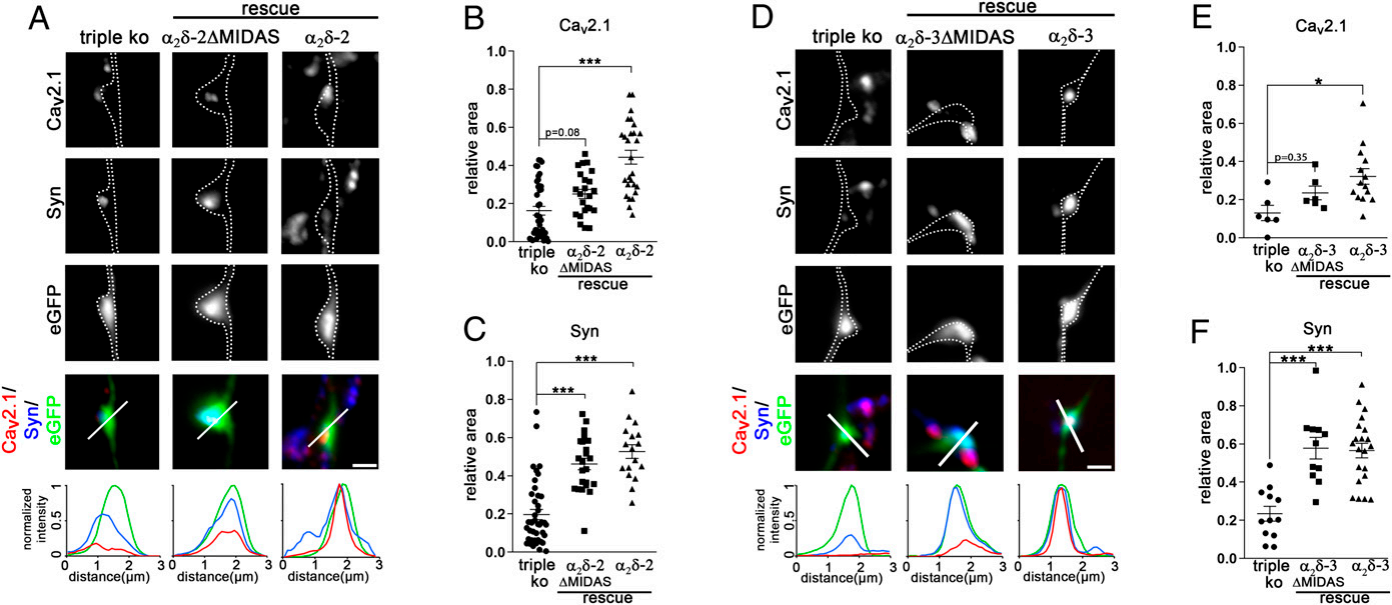
Rescuing triple-knockout/knockdown synapses with α_2_δ-2-ΔMIDAS or α_2_δ-3-ΔMIDAS dissociates synapse differentiation from presynaptic calcium channel trafficking. (*A* and *D*) Immunofluorescence micrographs of axonal varicosities from presynaptic α_2_δ TKO/KD neurons (triple KO, eGFP-positive axonal varicosities, *Left*) and neurons expressing α_2_δ-2-ΔMIDAS or α_2_δ-2 (*A*) and α_2_δ-3-ΔMIDAS or α_2_δ-3 (*D*). Axonal varicosities are outlined by a dashed line. Immunolabeling for Ca_V_2.1 and synapsin (syn) revealed that, unlike α_2_δ-2 or α_2_δ-3, expression α_2_δ-2-ΔMIDAS or α_2_δ-3-ΔMIDAS in TKO/KD neurons fully rescued presynaptic synapsin but not Ca_V_2.1 clustering. The relative fluorescence of each signal was recorded along the indicated line to support these observations. (*B*, *C*, *E*, and *F*) Quantification of the relative synaptic area covered by the respective immunofluorescence of presynaptic Ca_V_2.1 (*B* and *E*) and synapsin (*C* and *F*) [ANOVA, α_2_δ-2: Ca_V_2.1, *F*_(2,__78)_ = 18.9, *P* < 0.001, *n* = 41 (triple KO), 23 (MIDAS), and 17 (rescue) from 3 to 10 culture preparations; synapsin, *F*_(2,__56)_ = 18.7, *P* < 0.001, *n* = 19 (triple KO), 23 (MIDAS), and 17 (rescue) from three to four culture preparations; α_2_δ-3: Ca_V_2.1, *F*_(2,__23)_ = 4.7, *P* < 0.019, *n* = 6 (triple KO), 6 (MIDAS), and 14 (rescue) from two to three culture preparations; synapsin: *F*_(2,__41)_ = 17.3, *P* < 0.001, *n* = 12 (triple KO), 11 (MIDAS), and 21 (rescue) from three to four culture preparations; Tukey post hoc test, *P = 0.016, ***P < 0.001; horizontal lines represent means and error bars SEM]. (Scale bars, 1 µm.)

The severe consequences of presynaptic α_2_δ triple loss of function on pre- and postsynaptic composition and synaptic ultrastructure strongly suggest a functional redundancy. Thus, to further elucidate the potentially redundant roles of α_2_δ subunits in pre- and postsynaptic differentiation, we analyzed the propensity of each individual isoform in rescuing synapse formation and differentiation. First, α_2_δ-2/-3 double-knockout neurons which solely express α_2_δ-1 showed a proper apposition of pre- and postsynaptic proteins (see colocalized synaptic markers near the eGFP-positive TKO/KD axons indicated by asterisks in Figs. 1*C*, 3 *A* and *B*, and 4 *B* and *E*). Moreover, the α_2_δ TKO/KD phenotype could be rescued by the expression of α_2_δ-2 (rescue in Figs. 1 *D* and *E*, 3, 4*E*, and 7 and *SI Appendix*, Fig. S5), α_2_δ-1 (*SI Appendix*, Figs. S6 and S8), and α_2_δ-3 (Fig. 7 and *SI Appendix*, Fig. S8). Together this shows that the apparent critical roles of α_2_δ subunits in glutamatergic synapse formation are highly redundant between the neuronal α_2_δ isoforms.

### Expressing α_2_δ-ΔMIDAS Mutants in Triple-Knockout/Knockdown Neurons Fully Rescues Presynaptic Synapsin but Not Calcium Channel Clustering.

Our experiments demonstrate an essential role of α_2_δ subunits in glutamatergic synapse formation and differentiation which might be related to the failure of presynaptic calcium channel trafficking. Alternatively, however, α_2_δ subunits may act transsynaptically and independent of the calcium channel complex, as has been previously suggested (5, 13, 16, 18). α_2_δ subunits contain a von Willebrand factor type A (VWA) domain which, at least in α_2_δ-1 and α_2_δ-2, includes a perfect metal ion-dependent adhesion site (MIDAS). The integrity of the MIDAS motif in α_2_δ subunits is necessary for calcium current enhancement and channel trafficking (7, 34, 35). This finding is supported by the proposed structure of α_2_δ-1, in which the VWA domain and particularly the MIDAS are facing the surface of the pore-forming α_1_ subunit and are thus predicted to be crucial for α_1_ and α_2_δ subunit interactions (36). We reasoned that mutating the MIDAS site, which has previously been shown to inhibit channel trafficking (35), may be helpful in dissociating channel-dependent from potential channel-independent functions of α_2_δ subunits. To this end we mutated the amino acids D300, S302, and S304 of α_2_δ-2 (α_2_δ-2-ΔMIDAS) and D262, S264, and S266 of α_2_δ-3 (α_2_δ-3-ΔMIDAS) to alanines and analyzed to which extent expression of the ΔMIDAS mutants can rescue synaptic targeting of endogenous calcium channels and synapsin clustering. Both ΔMIDAS mutants are expressed at the cell surface and can cluster in presynaptic boutons, similar to wild-type α_2_δ-2 and α_2_δ-3 (*SI Appendix*, Fig. S9). While α_2_δ-2-ΔMIDAS (Fig. 7*A*) rescued presynaptic Ca_V_2.1 labeling only partially to 31% of the rescue observed with normal α_2_δ-2 (Fig. 7*B*), presynaptic synapsin labeling was almost fully rescued to 83% of α_2_δ-2 (Fig. 7*C*). Similarly, α_2_δ-3-ΔMIDAS (Fig. 7*D*) rescued presynaptic Ca_V_2.1 labeling partially to 55% (Fig. 7*E*), while presynaptic synapsin clustering was fully rescued to 104% of normal α_2_δ-3 (Fig. 7*F*). Taken together, expression of α_2_δ-ΔMIDAS mutants in α_2_δ TKO/KD synapses suggests that presynaptic synapsin accumulation and calcium channel trafficking are functions differentially mediated by α_2_δ subunits.

## Discussion

Many brain neurons simultaneously and abundantly express three different α_2_δ subunit isoforms (16, 27, 37), a fact, which, until today, has complicated studying their potentially redundant roles. By establishing a cellular α_2_δ subunit triple loss-of-function model, we here identified a critical and highly redundant role of presynaptic α_2_δ subunits in regulating glutamatergic synapse formation and differentiation, as evidenced by a series of observations. First, excitatory synapses from triple-knockout/knockdown cultures show a severe failure in activity-dependent FM-dye uptake. Second, lack of presynaptic α_2_δ subunits strongly reduces somatic calcium currents, presynaptic calcium transients, and clustering of endogenous P/Q-type (Ca_V_2.1) and N-type (Ca_V_2.2) calcium channels, and the size of the AZ. Third, the failure in presynaptic differentiation is accompanied by reduced clustering of postsynaptic AMPARs and thinning of the PSD. Fourth, the severe synaptic phenotype, particularly affecting presynaptic calcium channel expression, presynaptic calcium influx, and accumulation of presynaptic vesicles (based on synapsin and vGLUT1 labeling), can be rescued by the sole expression of α_2_δ-1, α_2_δ-2, or α_2_δ-3. Fifth, α_2_δ-2 and α_2_δ-3 with mutated MIDAS sites only partially rescue presynaptic calcium channel clustering although they fully rescue presynaptic synapsin expression, strongly supporting channel-independent presynaptic roles of α_2_δ subunits.

### Presynaptic α_2_δ Isoforms Redundantly Regulate Synaptic Differentiation of Glutamatergic Synapses.

An increasing number of studies over the recent years have implicated calcium channel α_2_δ subunits in synaptic functions (reviewed in refs. 5 and 33). However, the severity of the phenotype of specific α_2_δ loss-of-function models strongly correlated with the expression level of the particular isoform in the affected cells or tissues: Knockdown of α_2_δ-1 affected synapse formation in retinal ganglion cells (11, 12), lack of α_2_δ-2 causes pre- and postsynaptic defects in hair cells of the inner ear (13) and affected cerebellar climbing fiber synapses (15), knockout of α_2_δ-3 alters presynaptic morphology of auditory nerves (19), and in invertebrates loss of function of the homologous subunit resulted in abnormal presynaptic development in motoneurons (17, 18). Finally, the predominant expression of α_2_δ-4 in the retina (38) is mirrored by retinal defects and consequences on the organization of rod and cone photoreceptor synapses (20, 21, 39). Contrary to these specialized cell types and tissues, the mammalian brain expresses all four known α_2_δ isoforms (37, 40), whereby the isoforms α_2_δ-1, -2, and -3 are strongly and most ubiquitously expressed (16, 27). While the increasing severity of the phenotypes between α_2_δ subunit single and double-knockout mice already suggested a functional redundancy, this was ultimately revealed in the cellular triple-knockdown/knockout model established for the present study. This functional redundancy is a feature that is shared with the ubiquitous transsynaptic cell adhesion proteins neuroligin and neurexin (41, 42). In contrast to knockout animal models, in which the detailed cellular phenotypes may be masked by potential compensatory effects, for example by isoform redundancy or developmental adaptations, the present cellular triple-knockout/knockdown model allowed analyzing the consequences of a complete lack of α_2_δ subunits in neurons from the central nervous system. Thus, our study proves that presynaptic expression of α_2_δ subunits is critical for the proper development and differentiation of excitatory glutamatergic synapses. The functional redundancy of the three neuronal α_2_δ isoforms was particularly evident in rescuing the severe consequence on synapse formation. This does not, however, exclude isoform-specific differences in modulating specific synaptic or channel-dependent functions (43). For example, the sole presence of α_2_δ-1 could not fully rescue somatodendritic calcium current densities and the expression of α_2_δ-3 seemed to less strongly recruit presynaptic Ca_V_2.1 clustering (*SI Appendix*, Fig. S8). In contrast to excitatory glutamatergic synapses, GABAergic synapses could still form in the absence of α_2_δ subunits. This synapse specificity is particularly interesting as α_2_δ subunits are also critical regulators of inhibitory synapse connectivity. For example, we have recently identified that a single splice variant of the presynaptic α_2_δ-2 isoform transsynaptically regulates postsynaptic GABA-receptor abundance and synaptic wiring (16). Also, our present study finds a small but not significant reduction of presynaptic Ca_V_2.1 clustering in triple-knockout/knockdown GABAergic medium spiny neurons (MSNs) (Fig. 5*G*). Together this suggests that synapse formation and transsynaptic signaling are two independent functions of α_2_δ subunits. The exclusive dependence of glutamatergic synaptogenesis on presynaptically expressed α_2_δ subunits is supported by the recent finding that the antiepileptic and antiallodynic drug gabapentin prevents synaptogenesis between sensory and spinal cord neurons by acting on presynaptic α_2_δ-1 subunits (44).

### α_2_δ Subunits Are Critical Regulators of Synapse Formation.

**Fig. 8.**
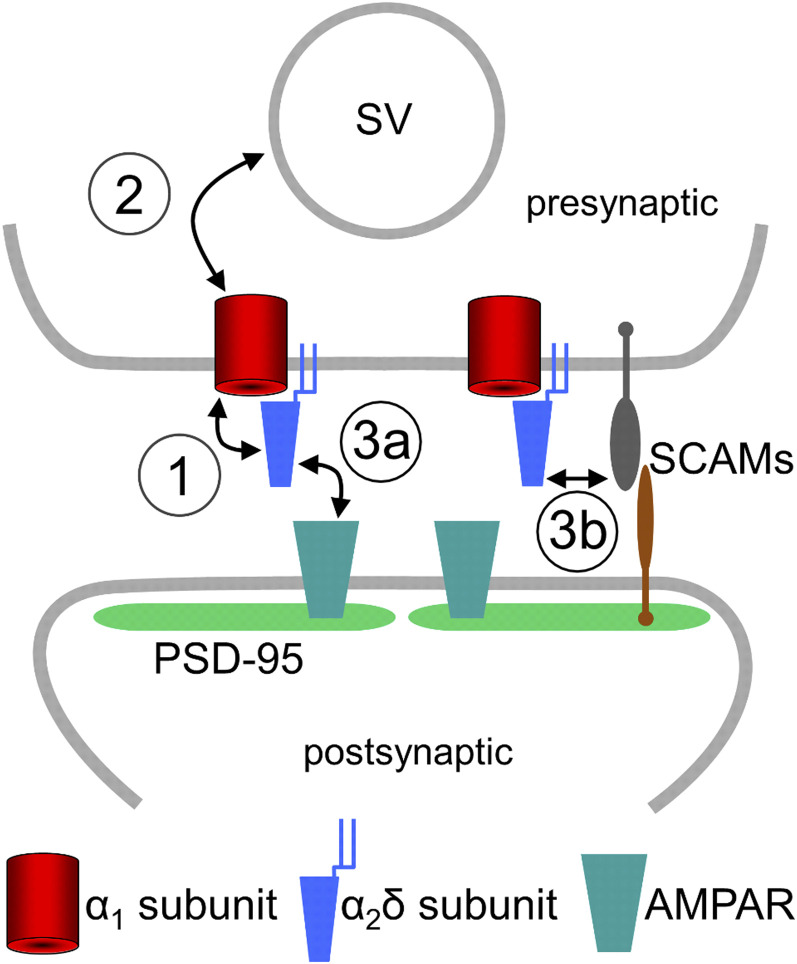
Model summarizing the putative roles of presynaptic α_2_δ subunits in glutamatergic synapse formation and differentiation. Our findings identified α_2_δ subunits as key organizers of glutamatergic synapses and propose their involvement in at least three critical steps during synapse maturation. By interacting with the α_1_ subunit they mediate the incorporation of VGCCs into the presynaptic AZ (1). α_2_δ subunits are involved in presynaptic differentiation and may, directly and/or indirectly via the entire VGCC complex, mediate the accumulation of synaptic vesicles (SV) to the synaptic terminal (2). Finally, α_2_δ subunits align the presynaptic AZ with the postsynaptic membrane and postsynaptic AMPARs. This may be mediated by a direct interaction with AMPARs (3a) or by interacting with classical synaptic cell adhesion molecules (SCAMs, 3b), as for example neurexins.

In general, synaptic cell adhesion molecules are thought to mediate the initial contact formation between axons and dendrites (45, 46). The vesicle-associated protein synapsin is an early marker for presynaptic vesicle recruitment (47), yet its accumulation fails in α_2_δ triple-knockout/knockdown neurons. Nevertheless, the presence of synapse-like axonal varicosities (*SI Appendix*, Fig. S4) reveals an intact axodendritic contact formation. This observed failure in synaptic vesicle recycling can thus be explained by two findings in our study. On the one hand, the marked reduction of presynaptic calcium channels (Fig. 3), somatodendritic calcium currents (Fig. 1), and, ultimately, presynaptic calcium transients (Fig. 2) suggests severely reduced presynaptic calcium influx. On the other hand, the strong reduction of presynaptic synapsin (Figs. 3 and 4) and vGLUT1 (Fig. 4*G* and *SI Appendix*, Fig. S6) suggests a major defect in the accumulation of presynaptic vesicles. Together this suggests that α_2_δ subunits and therefore probably VGCC complexes take a leading role in synaptogenesis: Without α_2_δ subunits excitatory synapses fail to differentiate and mutation of the MIDAS motif prevents normal presynaptic calcium channel trafficking but not synapsin accumulation. Previous models suggested that VGCC complexes are secondarily recruited to the release sites via their manifold interactions with presynaptic proteins (45, 48). Our findings also support the hypothesis that extracellular α_2_δ subunits organize the alignment of the presynaptic AZ with the PSD. Indeed, the published extracellular structure of α_2_δ-1 of the skeletal muscle Ca_V_1.1 complex (36) proposes the protrusion of α_2_δ subunits far into the synaptic cleft. Thus, α_2_δ subunits may couple calcium channels with postsynaptic receptors, thereby aligning the presynaptic AZ with the PSD. This hypothesis is supported by the observation that in the auditory hair-cell synapse postsynaptic AMPAR clusters are dispersed in α_2_δ-2 knockout mice (13) and that presynaptic α_2_δ-2 regulates postsynaptic GABA-receptor abundance in GABAergic synapses (16). Extracellular binding of α_2_δ subunits to the α_1_ subunit has been shown to be critical for efficiently coupling VGCCs to exocytosis (7). However, whether α_2_δ subunits interact directly or indirectly with postsynaptic receptors or transsynaptic linkers has yet to be elucidated. Evidently, synaptic cell adhesion molecules could provide potential candidates for such interactions (Fig. 8, point 3b). In this context it is noteworthy that α-neurexins, although not critical for synapse formation, link presynaptic calcium channels to neurotransmitter release via extracellular domains (41, 49) and regulate presynaptic Ca_V_2.1 channels via α_2_δ subunits (8). Interestingly, a recently established triple knockout of all three Cav2 subunits in cultured hippocampal neurons and at the calyx of Held abolished evoked exocytosis; however, synapse and AZ structure, vesicle docking, the transsynaptic organization, and localization of α_2_δ-1 were not impaired (50). Taken together, the identification of α_2_δ subunits as the first proteins that are absolutely critical for glutamatergic synapse formation paves the way for identifying up- and downstream interaction partners and signaling mechanisms.

### Proposed Synaptic Roles for α_2_δ Subunits and Future Implications.

Our study suggests an involvement of presynaptic α_2_δ subunits in several steps during synaptogenesis and synapse differentiation (Fig. 8). First, α_2_δ subunits mediate presynaptic calcium channel trafficking (Fig. 8, point 1), a role which was to be expected and which was previously demonstrated (51). Second, α_2_δ subunits are involved in presynaptic differentiation (Fig. 8, point 2). This becomes evident by the strong effect of triple knockout/knockdown on the accumulation of synaptic vesicle-associated proteins such as synapsin and the vesicular glutamate transporter vGLUT1. Although it is feasible that the manifold interaction sites within the intracellular loops of calcium channel α_1_ subunits link the channel complex to synaptic vesicles (48), the only partial rescue observed with the MIDAS mutations rather favors a role of α_2_δ subunits independent of the channel complex, as previously suggested (18). An in-depth analysis of these particular questions in our present study was impeded by the low availability of triple-knockout/knockdown cultures and the necessity for preembedding immunolabeling with the silver-amplified gold approach to visualize the sparsely distributed, featureless boutons in electron microscopy. Thus, elucidating the precise underlying molecular mechanisms requires the future development of novel experimental tools. Third, and as discussed above, α_2_δ subunits regulate postsynaptic receptor clustering and differentiation of the PSD and thus either directly or indirectly act as transsynaptic organizers (Fig. 8, points 3a and 3b). As suggested by previous studies (8, 16, 41, 49), certain functions of α_2_δ may be modulated by their interaction with classical synaptic cell adhesion molecules.

Taken together, our experiments identified a critical and redundant role of presynaptic α_2_δ subunits in glutamatergic synapse formation and differentiation. This affects our current view on excitatory synapse formation and implicates α_2_δ subunits and therefore presynaptic calcium channel complexes as potential nucleation points for the organization of synapses. Finally, these findings may provide a basis for understanding the role of α_2_δ subunits in the development and clinical manifestation of neuropsychiatric disorders (52, 53).

## Materials and Methods

A more detailed description of the materials and methods can be found in *SI Appendix*.

### Breeding and Genotyping of α_2_δ-2/-3 Double-Knockout Mice.

Double-knockout mice and littermate controls were obtained by cross-breeding double-heterozygous α_2_δ-3^+/−^, α_2_δ-2^+/du^ mice, both back-crossed into a c57BL/6N background for more than 10 generations (*SI Appendix*). Animal protocols, including breeding of single- and double-knockout mice, were approved by the Austrian Federal Ministry of Science, Research and Economy (BMWFW-66.011/0113-WF/V/3b/2014, BMWFW-66.011/0114-WF/V/3b/2014, 2020-0.121.342, and 2020-0.107.333). The number of animals used for this project was annually reported to the Austrian Federal Ministry of Science, Research and Economy (BMWFW).

### Quantitative TaqMan Copy Number RT-PCR.

In order to ultimately confirm the genomic duplication of the Cacna2d2 gene in α_2_δ-2^du/du^ mice we developed a custom-designed copy number (CN) qPCR assay (*SI Appendix*, Fig. S3).

### Primary Cultured Hippocampal Neurons.

Low-density cultures of hippocampal neurons were prepared from putative P0-P3 du/α_2_δ-3 double-knockout mice and littermate controls as described previously (54–57). For electrophysiology neurons were plated directly on top of glial cells as previously reported (58).

### Primary Cocultures of Striatal and Cortical Neurons and Transfection Procedure.

Cocultures of GABAergic striatal MSNs and glutamatergic cortical neurons were prepared from P0 to P3 du/α_2_δ-3 double-knockout mice and littermate controls (α_2_δ-3 knockout) as described previously (16) (for details see *SI Appendix*). Cells were processed for immunostaining at 22 to 24 d in vitro (DIV).

### Transfection of Hippocampal Neurons.

Expression plasmids were introduced into neurons at 6 DIV using Lipofectamine 2000-mediated transfection (Invitrogen) as described previously (56). α_2_δ TKO/KD cultures were established by employing pβA-eGFP-U6-α_2_δ-1-shRNA (30) knockdown in α_2_δ-2(du)/α_2_δ-3 double-knockout neurons. Littermate controls were transfected with pβA-eGFP. For cotransfection/rescue experiments (pβA-eGFP-U6-α_2_δ-1-shRNA plus pβA-α_2_δ-2 or pβA-α_2_δ-3) 1.5 μg of total DNA was used at a molar ratio of 1:2, respectively. Cells were processed for patch clamp experiments and immunostaining/FM-dye loading at 14 to 16 DIV and 17 to 25 DIV, respectively, after plating.

### Molecular Biology.

To facilitate neuronal expression all constructs were cloned into a eukaryotic expression plasmid containing a neuronal chicken β-actin promoter, pβA (59). Cloning of all constructs was confirmed by sequencing (Eurofins Genomics) and sequences were deposited in GenBank. Detailed cloning procedures are available in *SI Appendix*, *Supplementary Materials and Methods*).

### Electrophysiology.

Calcium channel activity was recorded using the whole-cell patch-clamp technique as described previously (58) with modifications (*SI Appendix*).

### FM-Dye Loading.

Live cell DIV 17 to 25 cultured hippocampal neurons were preincubated in 2.5 mM KCl Tyrode solution in a specialized Ludin chamber (Life Imaging services) (60). To block network activity 10 μM CNQX and 50 μM AP5 (both Tocris Bioscience) were present in all solutions and the temperature was kept at 37 °C. Cells were loaded with FM4-64 dyes upon 60 mM KCl depolarization followed by a continuous washout with Tyrode solution (2.5 mM KCl) using an inverted Axiovert 200 M setup (Carl Zeiss Light Microscopy) connected to a Valve Link perfusion system. Quantification and analysis are described in *SI Appendix*.

### Calcium Imaging.

Presynaptic calcium transients were visualized using GCaMP6f coupled to synaptophysin driven by a synapsin promotor as previously described (8, 32) (*SI Appendix*).

### Immunocytochemistry.

Immunolabeling of permeabilized neurons was performed as previously described (61) (for details see *SI Appendix*). Coverslips were observed with an Axio Imager microscope (Carl Zeiss) using 63×, 1.4 numerical aperture (NA) oil-immersion objective lens or with an Olympus BX53 microscope using a 60×, 1.42 NA oil-immersion objective lens. Images were recorded with cooled charge-coupled device cameras (SPOT Imaging Solutions and XM10; Olympus).

### Electron Microscopy.

Cultures of neurons were prepared as described above with the exception that neurons were grown on coverslips coated with a carbon layer as previously described (62) and fixed with 2% glutaraldehyde (Agar Scientific Ltd.) in phosphate buffer (0.1 M, pH 7.4). Detailed procedures for structural analysis and preembedding immuoelectron microscopy are presented in *SI Appendix*.

### Antibodies.

Details on primary and secondary antibodies are presented in *SI Appendix*.

### Analysis and Quantification.

Synaptic expression of α_2_δ isoforms, synaptic colocalization, single bouton quantification, and analysis of electron micrographs were performed using MetaMorph (Molecular Devices) or ImageJ (NIH; https://imagej.net/ImageJ) software, partly by using custom-built macros. Details on analysis procedures as well as analyses of electrophysiological recordings and presynaptic calcium transients are outlined in *SI Appendix*, *Supplementary Materials and Methods*. Further data analysis was performed with MS Excel and Graph Pad Prism, and image composites were arranged in Photoshop CS6 and Affinity Photo.

### Statistical Analysis.

Results are expressed as means ± SEM except where otherwise indicated. The type of statistical test used is given in the respective figure legends. The statistical requirements and assumptions underlying each test were evaluated for each data set (e.g., data normality, independence, similarity of variances, etc.) and, if violated, and alternative nonparametric test was selected for the analysis as indicated. Data were organized and analyzed using MS Excel and Graph Pad Prism (Graph Pad Software, La Jolla, CA, USA). Graphs and figures were generated using Graph Pad Software, Adobe Phostshop CS6, and Affinity Photo.

## Supplementary Material

Supplementary File

Supplementary File

Supplementary File

## Data Availability

All study data are included in the article and/or supporting information. Some study data are available upon request.
