# Supplementary material for: Presynaptic α2δ subunits are key organizers of glutamatergic synapses
Source: Proc Natl Acad Sci U S A. 2021 Mar 29;118(14):e1920827118. doi: 10.1073/pnas.1920827118 (PMC8040823; doi:10.1073/pnas.1920827118)
Supplement: Supplementary File [file pnas.1920827118.sapp.pdf]

PNAS
www.pnas.org

Supplementary Information for

Presynaptic $\alpha_2\delta$ subunits are key organizers of glutamatergic synapses.

Clemens L. Schöpf, Cornelia Ablinger, Stefanie M. Geisler, Ruslan I. Stanika, Marta Campiglio, Walter A. Kaufmann, Benedikt Nimmervoll, Bettina Schlick, Johannes Brockhaus, Markus Missler, Ryuichi Shigemoto, and Gerald J. Obermair

Address correspondence to:
Gerald J. Obermair
Email: Gerald.Obermair@kl.ac.at

This PDF file includes:

Supplementary Materials and Methods
Figures S1 to S9
Table S1
Legends for Movies S1 to S2
SI References

Other supplementary materials for this manuscript include the following:

Movies S1 to S2

Supplementary Materials and Methods

Breeding of α2δ-2/-3 double-knockout mice. Double-knockout mice and littermate controls were obtained by crossbreeding double heterozygous $\alpha_2\delta$-3$^{+/-}$, $\alpha_2\delta$-2$^{+/du}$ mice, both backcrossed into a c57BL/6N background for more than 10 generations. $\alpha_2\delta$-3 knockout (Cacna2d3^{tm1Dgen}) strains generated by Deltagen (San Mateo, CA, USA) and ducky (Cacna2d2^{du}; $\alpha_2\delta$-2$^{du/du}$) mice were obtained from The Jackson Laboratory (Bar Harbor, ME, USA). One week before delivery, male mice were separated from the breeding cages and BALB/c foster mothers were included. Mice were bred and maintained at the central laboratory and animal facility of the Medical University Innsbruck according to national and EU regulations and conforming to the Austrian guidelines on animal welfare and experimentation. Animal protocols, including breeding of single- and double-knockout mice, were approved by the Austrian Federal Ministry of Science, Research and Economy (BMWFW-66.011/0113-WF/V/3b/2014, BMWFW-66.011/0114-WF/V/3b/2014, 2020-0.121.342, and 2020-0.107.333). Survival rate of different α2δ double-knockout pups was continuously monitored over 3 years, whereby the guidelines for identifying humane endpoint criteria were strictly applied. The number of animals used for this project was annually reported to the Austrian Federal Ministry of Science, Research and Economy (bmwfw).

Tattooing and genotyping of potential double-knockout mice. To identify mice for genotyping, newborn pups were tattooed on the paws using a sterile needle with green tattoo paste (ketschum.mfg.co).

Genotyping. DNA was extracted by incubating a tail biopsy of ~1-2 mm length in 100 µl of 25 mM NaOH at 95 °C for 30 min followed by cooling to 4 °C and the addition of 100 µl of 40 mM Tris-HCl neutralization buffer. The PCR reaction buffer further contained 1.25 mM MgCl, 0.125 mM dNTP's, 1 mM 5x Green GoTaq Flexi Buffer, 0.5 mM Green GoTaq Polymerase (Promega) and 2 µl of genomic DNA. Probes were analyzed using standard PCR conditions and gel electrophoresis. *Cacna2d3^{tm1Dgen} ($\alpha_2\delta$-3 knockout mice):* The following primers were used for detecting the wildtype allele (F1-R, 183 bp fragment) and the knockout allele (F2-R, 331 bp fragment): F1:5'-TAGAAAAGATGCACTGGTCACCAGG-3'; F2: 5'-GGGCCAGCTCATTCCTCCCACTCAT-3', R: 5'-GCAGAAGGCACATTGCCATACTCAC-3'. *Cacna2d2^{du} ($\alpha_2\delta$-2$^{du/du}$, ducky mice):* The evaluation of the ducky mutation was performed as previously described (1). PCR with the primers du-F, 5'-ACCTATCAGGCAAAAGGACG-3' and du-R 5'-AGGGATGGTGATTGGTTGGA-3' revealed a fragment of 541 bp for both the wildtype and the du allele from a region that is duplicated in the du allele. Digestion with BspHI (New England Biolabs) resulted in two fragments of 286 bp and 273 bp for the du allele, whereas the fragment from the wild-type allele remained uncut. Wildtype mice could be identified by the presence of a single band upon agarose gel electrophoresis. Heterozygous and homozygous (du/du) mice each showed two bands and were preliminarily distinguished based on the relative intensity of the double band.

Quantitative TaqMan copy number RT-PCR. In order to ultimately confirm the genomic duplication of the Cacna2d2 gene in $\alpha_2\delta$-2$^{du/du}$ mice we developed a custom designed copy number (CN) qPCR assay. Tissue biopsies of putative knockout and littermate controls were incubated at 55°C overnight constantly shaking at 550 rpm, using 250 µl Direct PCR Tail Lysis reagent (PeqLab) and 2.5 µl Protease K (20mg/ml, Roche). Following incubation, Protease K was inactivated by incubation at 85°C for 45 min constantly shaking at 550 rpm and subsequently DNA content was measured using a NanoDrop 2000 Spectrophotometer (Thermo Scientific). For each reaction 8 µl DNA (5 ng/µl) together with 10 µl TaqMan Mastermix, 1 µl Cacna2d2 CN assay labeled with a FAM dye (assay ID: Mm00270662-cn), and 1 µl transferrin receptor (Tfrc) assay as reference gene

containing a VIC dye (catalogue number 4458366) were used. Chemicals were purchased from Thermo Fisher Scientific and samples were analyzed in triplicates with a 7500 fast real time PCR machine (Thermo Fisher Scientific). Relative gene expression was calculated by using the $^{\Delta\Delta}C_T$-method (2) normalized to wildtype control samples yielding ratios of 1 for wildtype samples (2 alleles), 1.5 for heterozygous samples (3 alleles), and 2 for homozygous ducky samples (4 alleles) (see Fig. S3). The abundance of different $\alpha_2\delta$ subunit transcripts in cDNA from cultured hippocampal neurons or hippocampus tissue was assessed by TaqMan quantitative PCR (qPCR) using a standard curve method as previously described (3). TaqMan gene expression assays specific for the four $\alpha_2\delta$ isoforms were designed to span exon–exon boundaries and purchased from Applied Biosystems. The following assays were used [name (gene symbol), assay ID (Applied Biosystems)]: $\alpha_2\delta$-1 (Cacna2d1), Mm00486607_m1; $\alpha_2\delta$-2 (Cacna2d2), Mm00457825_m1; $\alpha_2\delta$-3 (Cacna2d3), Mm00486613_m1; $\alpha_2\delta$-4 (Cacna2d4), Mm01190105_m1. Expression of hypoxanthine phosphoribosyl-transferase 1 (HPRT1; Mm00446968_m1) was used as endogenous control. The qPCR (50 cycles) was performed in duplicates using total cDNA and the specific TaqMan gene expression assay for each 20 µl reaction in TaqMan Universal PCR Master Mix (Applied Biosystems). Analyses were performed using the 7500 Fast System (Thermo Fisher Scientific). The Ct values for each gene expression assay were recorded in each sample and molecule numbers were calculated from the respective standard curve (3). Expression of HPRT1 was used to evaluate the total mRNA abundance and for normalization to allow a direct comparison between the expression levels in the different genotypes.

Primary cultured hippocampal neurons. Low-density cultures of hippocampal neurons were prepared from putative P0-P3 du/$\alpha_2\delta$-3 double-knockout mice and littermate controls as described previously (4-7). Briefly, dissected hippocampi were dissociated by trypsin treatment and trituration. For imaging experiments neurons were plated on poly-L-lysine-coated glass coverslips in 60 mm culture dishes at a density of ~3500 cells/cm^2. After plating, cells attached for 3-4 h before transferring the coverslips neuron-side down into a 60 mm culture dish with a glial feeder layer. For electrophysiology neurons were plated directly on top of glial cells as previously reported (8). Neurons and glial feeder layer were maintained in serum-free neurobasal medium supplemented with Glutamax and B-27 (all from Invitrogen). Ara-C (5µM) was added 3d after plating and, once a week, 1/3 of the medium was removed and replaced with fresh maintenance medium.

Primary co-cultures of striatal and cortical neurons and transfection procedure. Co-cultures of GABAergic striatal medium spiny neurons (MSNs) and glutamatergic cortical neurons were prepared from P0-P3 du/$\alpha_2\delta$-3 double-knockout mice and littermate controls ($\alpha_2\delta$-3 knockout) as described previously (9). Briefly, striatal and cortical tissue of each pup was separately collected and dissociated by trypsin treatment and trituration. Subsequently, expression plasmids were introduced into MSNs using Lipofectamine 2000-mediated transfection (Invitrogen) as described previously (6). To this end, ~2.4x10^5 striatal neurons were transfected for 20 min in a 37°C water bath keeping the total volume to ≥ 1 ml with NBKO. Triple-knockout MSNs were generated by employing pβA-eGFP-U6-$\alpha_2\delta$-1-shRNA (10) knock-down in du/$\alpha_2\delta$-3 double-knockout neurons. Littermate controls were transfected with pβA-eGFP, yielding $\alpha_2\delta$-3 knockout neurons in which $\alpha_2\delta$-1 and $\alpha_2\delta$-2 are thus still present. After 20 min the cell suspension was directly seeded on poly-L-lysine coated glass coverslips within a 60 mm culture dish containing 4 ml of pre-warmed NPM and striatal neurons were allowed to attach at 37°C. For the entire transfection procedure, dissociated cortical neurons were maintained in HBSS in a 15 ml tube at 37°C and occasionally swirled. After 2 h transfection of striatal neurons was stopped by replacing the transfection-plating solution with

5 ml of fresh, pre-warmed NPM and untransfected cortical neurons were seeded onto striatal neurons in a ratio of 2 (cortical neurons) to 3 (MSNs) at a total density of ~14,000 cells/cm^2. Subsequently, cortical cells were allowed to attach for 3–4 h until coverslips were transferred neuron-side down into a 60 mm culture dish containing a glial feeder layer. Ara-C treatment and maintenance of neurons and glia was done as described above. Cells were processed for immunostaining at 22-24 DIV.

Transfection of hippocampal neurons. Expression plasmids were introduced into neurons at 6 days in vitro using Lipofectamine 2000-mediated transfection (Invitrogen) as described previously (6). Triple-knockout cultures were established by employing pβA-eGFP-U6-$\alpha_2\delta$-1-shRNA (10) knock-down in du/$\alpha_2\delta$-3 double-knockout neurons. Littermate controls were transfected with pβA-eGFP. For co-transfection/rescue experiments (pβA-eGFP-U6-$\alpha_2\delta$-1-shRNA plus pβA-$\alpha_2\delta$-2 or pβA-$\alpha_2\delta$-3) 1.5 µg of total DNA was used at a molar ratio of 1:2, respectively. Cells were processed for patch clamp experiments and immunostaining/FM-dye loading at 14-16 DIV and 17-25 DIV, respectively after plating.

Molecular biology. To facilitate neuronal expression all constructs were cloned into a eukaryotic expression plasmid containing a neuronal chicken β-actin promoter, pβA (11). Cloning of all constructs was confirmed by sequence (Eurofins Genomics, Germany) and sequences were deposited in Genbank. Functionality of HA epitope-tagged $\alpha_2\delta$ subunits (see below) based on heterologous co-expression with calcium channel α_1 subunits in HEK cells was routinely confirmed by electrophysiology and electrophysiological characterization of HA epitope-tagged $\alpha_2\delta$-1 and $\alpha_2\delta$-3 was previously published (12). 2HA-$\alpha_2\delta$-2 and $\alpha_2\delta$-1 tagged with super-ecliptic pHluorin (SEP, see below) were tested by recording whole-cell calcium currents in tsA201 cells. Half-maximal activation potential of $Ca_V2.1+\beta_{4e}$ was not different upon co-expression of HA-tagged compared to untagged $\alpha_2\delta$-2 ($V_{0.5}$: -1.3±0.7 vs. -3.0±1.3 mV±SEM, p=0.29, $t_{(14)}$=1.1). In contrast, half-maximal activation potential of $Ca_V1.3_{42}+\beta_3$ calcium currents was right-shifted upon co-expressing SEP-$\alpha_2\delta$-1 when compared to untagged $\alpha_2\delta$-1 ($V_{0.5}$: +3.2±3.6 vs. -8.7±0.7 mV±SEM, p<0.001, $t_{(26)}$=5.3).

pβA-$\alpha_2\delta$-1-v2: Mouse $\alpha_2\delta$-1 was cloned from genomic cDNA derived from mouse cerebellum. Primer sequences were selected according to Genbank NM-001110844. Briefly, the cDNA of $\alpha_2\delta$-1 was amplified by PCR in three fragments. The forward primer used for amplifying fragment 1 introduced a NotI site and the Kozak sequence (CCTACC) upstream the starting codon and the reverse primer used for amplifying fragment 3 introduced a KpnI and a SalI site after the stop codon. Fragment 2 (nt 1442-2564) was MfeI/BamHI digested and fragment 3 (nt 2335-3276) was KpnI/BamHI digested and co-ligated in the corresponding MfeI/KpnI sites of the pβA vector, yielding an intermediate construct. Fragment 1 (nt 1-1575) was NotI/MfeI digested and co-ligated with the SalI/MfeI digested intermediate construct, containing fragment 2 and 3, and the NotI/SalI digested pβA vector, yielding pβA-$\alpha_2\delta$-1-v2 (Genbank accession number MK327276; (9)).

pβA-2HA-$\alpha_2\delta$-1-v2: The putative signal peptide (aa1-24) was predicted using Signal P (SignalP 4.0: discriminating signal peptides from transmembrane regions) (13). A double HA tag (2HA) followed by a TEV cleavage site was introduced between the third and fourth amino acids after the predicted signal peptide cleavage site of mouse $\alpha_2\delta$-1, i.e. residue F27. Introduction of this sequence did not alter the predicted cleavage site. Briefly the cDNA sequence of $\alpha_2\delta$-1 (nt 1–516) was PCR amplified with overlapping primers introducing the double HA tag and the TEV cleavage site in separate PCR reactions using pβA-$\alpha_2\delta$-1 as template. The two separate PCR products were then used as templates for a final PCR reaction with flanking primers to connect the nucleotide sequences. The

resulting fragment was then NotI/BglII digested and ligated into the corresponding sites of pβA-$\alpha_2\delta$-1, yielding pβA-2HA-$\alpha_2\delta$-1-v2 (9).

pβA-SEP-$\alpha_2\delta$-1: $\alpha_2\delta$-1 (Genbank accession No. M21948 (14)) was cloned into pβA and the GFP variant super-ecliptic pHluorin (SEP) was inserted after the signal sequence (after aa 26) yielding pβA-SEP-$\alpha_2\delta$-1.

pβA-$\alpha_2\delta$-2-v1: Mouse $\alpha_2\delta$-2 was cloned from genomic cDNA from mouse brain. Primer sequences were selected according to Genebank NM-001174047. The cDNA of $\alpha_2\delta$-2 was amplified by PCR in 4 fragments. The forward primer used for amplifying fragment 1 introduced a HindIII site and the Kozak sequence (CCTACC). Fragment 1 was isolated from cerebellum, while the other three fragments from hippocampus. Fragment 1 (nt 1-686) and Fragment 2 (nt 323-1294) were HindIII/BamHI and BamHI/EcoRI digested respectively, and co-ligated in the corresponding HindIII/EcoRI sites of the pBS (Bluescript) vector, yielding the intermediate construct pBS- $\alpha_2\delta$-2-part1. Fragment 3 (nt 1137-2359) was EcoRI/PmlI digested and ligated into the corresponding sites of the pSPORT vector, yielding the intermediate construct pSPORT- $\alpha_2\delta$-2-part2. Fragment 4 (nt 2226-3444) was BmtI/XbaI digested and ligated into the corresponding sites of pSPORT-part2, yielding the intermediate construct pSPORT- $\alpha_2\delta$-2-part3. pSPORT-$\alpha_2\delta$-2-part3 was EcoRI/XbaI digested and the band containing fragments 3-4 (bp 1137-3444) was ligated into pBS-$\alpha_2\delta$-2-part1, yielding pBS-$\alpha_2\delta$-2. This construct was then HindIII/XbaI digested and the cDNA of $\alpha_2\delta$-2 was ligated into the pβA vector, yielding pβA-$\alpha_2\delta$-2-v1 (Genbank accession number MK327277; (9)).

pβA-2HA-$\alpha_2\delta$-2-v1: A putative signal peptide was not reliably predicted using Signal P (SignalP 4.0: discriminating signal peptides from transmembrane regions), (13) however the highest prediction showed that the signal peptide comprises residues 1-64. The 2HA tag followed by a thrombin cleavage site was therefore introduced after the predicted signal peptide cleavage site of mouse $\alpha_2\delta$-2, i.e. residue A64. Introduction of this sequence did not alter the predicted cleavage site. Briefly the cDNA sequence of $\alpha_2\delta$-2 (nt 1–761) was PCR amplified with overlapping primers introducing the double HA tag and the thrombin cleavage site in separate PCR reactions using pβA-$\alpha_2\delta$-2 as template. The two separate PCR products were then used as templates for a final PCR reaction with flanking primers to connect the nucleotide sequences. The resulting fragment was then HindIII/AflII digested and ligated into the corresponding sites of pβA-$\alpha_2\delta$-2, yielding pβA-2HA-$\alpha_2\delta$-2-1 (9).

pβA-2HA-$\alpha_2\delta$-2-ΔMIDAS: The three divalent metal-coordinating amino acids (D300, S302, and S302) of the MIDAS domain of α2δ-2 were mutated to alanines by SOE-PCR. Briefly the cDNA sequence of 2HA-$\alpha_2\delta$-2 (nt 1–1369) was PCR amplified with overlapping primers mutating the three amino acids into alanines in separate PCR reactions using pβA-2HA-$\alpha_2\delta$-2 as template. The two separate PCR products were then used as templates for a final PCR reaction with flanking primers to connect the nucleotide sequences. The resulting fragment was then HindIII/EcoRI digested and co-ligated with HindII/XbaI and XbaI/EcoRI fragments of pβA-2HA-$\alpha_2\delta$-2, yielding pβA-2HA-$\alpha_2\delta$-2-ΔMIDAS.

pβA-$\alpha_2\delta$-2-ΔMIDAS: The $\alpha_2\delta$-2 construct in which the three divalent metal-coordinating amino acids (D300, S302, and S302) of the MIDAS domain were mutated to alanines was obtained by simple restriction enzyme cloning. Briefly the N-terminal cDNA sequence of $\alpha_2\delta$-2 (nt 1-801) was isolated from pβA-$\alpha_2\delta$-2-v1 by digestion with HindIII/PflMI. The resulting fragment was ligated into the corresponding sites of pβA-2HA-$\alpha_2\delta$-2-*Δ*MIDAS, yielding pβA-$\alpha_2\delta$-2-*Δ*MIDAS.

pβA-$\alpha_2\delta$-3: Mouse $\alpha_2\delta$-3 was cloned from genomic cDNA from mouse hippocampus. Primer sequences were selected according to Genebank NM-009785. Briefly, the cDNA of $\alpha_2\delta$-3 was

amplified by PCR in four fragments. The forward primer used for amplifying fragment 1 introduced a NotI site and the Kozak sequence (CCTACC) upstream the starting codon. Fragment 3 (nt 1520-2817) was then SacI/PstI digested and fragment 4 (nt 2727-3276) was DraI/PstI digested and co-ligated in the corresponding SacI/SmaI sites of the pSPORT vector, yielding an intermediate construct. Fragment1 (nt 1-653) was then NotI/BamHI digested and fragment2 (535-1636) was then SacI/BamHI digested and co-ligated with the SacI/NotI digested intermediate construct, containing fragment3 and 4, yielding pSPORT-α$_2$δ-3. The cloned cDNA of α$_2$δ-3 was then NotI/RsrII digested and ligated into the corresponding sites of the pβA vector, yielding pβA-α$_2$δ-3 (Genbank accession number MK327280; (9)).

pβA-2HA-α$_2$δ-3: The putative signal peptide (aa 1-28) was predicted using Signal P (SignalP 4.0: discriminating signal peptides from transmembrane regions) (13). The 2HA tag followed by a thrombin cleavage site was therefore introduced after the predicted signal peptide cleavage site of mouse α$_2$δ-3, i.e. residue D28. Introduction of this sequence did not alter the predicted cleavage site. Briefly the cDNA sequence of α$_2$δ-3 (nt 1–653) was PCR amplified with overlapping primers introducing the double HA tag and the thrombin cleavage site in separate PCR reactions using pβA-α$_2$δ-3 as template. The two separate PCR products were then used as templates for a final PCR reaction with flanking primers to connect the nucleotide sequences. The resulting fragment was then NotI/BsrGI digested and ligated into the corresponding sites of pβA-α$_2$δ-3, yielding pβA-2HA-α$_2$δ-3 (9).

pβA-2HA-α$_2$δ-3-ΔMIDAS: The three divalent metal-coordinating amino acids (D262, S264, and S266) of the MIDAS domain of α$_2$δ-3 were mutated to alanines by SOE-PCR. Briefly the cDNA sequence of 2HA-α$_2$δ-3 (nt 1–1425) was PCR amplified with overlapping primers mutating the three amino acids into alanines in separate PCR reactions using pβA-2HA-α$_2$δ-3 as template. The two separate PCR products were then used as templates for a final PCR reaction with flanking primers to connect the nucleotide sequences. The resulting fragment was then NotI/BglII digested and ligated into the corresponding sites of pβA-2HA-α$_2$δ-3, yielding pβA-2HA-α$_2$δ-3-ΔMIDAS.

pβA-α$_2$δ-3-ΔMIDAS: The α$_2$δ-3 construct in which the three divalent metal-coordinating amino acids (D262, S264, and S266) of the MIDAS domain were mutated to alanines was obtained by simple restriction enzyme cloning. Briefly the N-terminal cDNA sequence of α$_2$δ-3 (nt 1-534) was isolated from pβA-α$_2$δ-3 by digestion with NotI/BsrGI. The resulting fragment was ligated into the corresponding sites of pβA-2HA-α$_2$δ-3-*Δ*MIDAS, yielding pβA-α$_2$δ-3-*Δ*MIDAS.

pβA-2HA-α$_2$-3-Δδ: A stop codon was introduced into the predicted site of cleavage between α$_2$ and δ peptides, according to Qin et al. (15), and the cDNA sequence encoding for the δ subunit was deleted by PCR. Briefly the cDNA sequence of 2HA-α$_2$δ-3 (nt 1254–2943) was PCR amplified with a reverse primer introducing a stop codon after residue T956. The resulting fragment was then BglII/RsrII digested and ligated into the corresponding sites of pβA-2HA-α$_2$δ-3, yielding pβA-2HA-α$_2$-3-Δδ.

pβA-eGFP-U6-shRNA-α$_2$δ-1: In brief siRNA target sequences corresponding to the α$_2$δ-1 coding region (Cacna2d1, GenBankTM accession number M_009784, see (10)) were selected and tested for efficient knockdown. The siRNA was expressed as shRNA under the control of a U6 promoter (derived from the pSilencer1.0-U6 siRNA expression vector, Ambion Ltd., Huntington, Cambridgeshire, UK) cloned into pβA-eGFP plasmid. For lentiviral expression α$_2$δ-1 shRNA was cloned into pHR as previously described (16).

pβA-eGFP: As described previously (6) enhanced green fluorescent protein (eGFP) was expressed from a eukaryotic expression plasmid containing a chicken β-active (BA) promoter (11).

pHR-βA-mcherry: As previously described (9), the coding sequence of mcherry (GeneBankTM accession number AKA95311.1) was inserted into a custom-built destination vector, pHR-βA-DEST, using LR Clonase II enzyme mixture (GATEWAY; Invitrogen).

pHR-βA-mcherry-U6-shRNA-α2δ-1: U6-shRNA-α2δ-1 was cloned into the pβA-mcherry plasmid. For the description of the shRNA construct pβA-eGFP-U6-shRNA-$\alpha_2\delta$-1see above.

SynGCaMP6f: Generation of synaptophysin-GCaMP6f driven by the synapsin promotor (synGCaMP6f) was previously described (17).

Electrophysiology.

Measurement of whole cell calcium currents: Calcium channel activity was recorded using the whole-cell patch-clamp technique as described previously (8) with modifications. Patch pipettes were pulled from borosilicate glass (Harvard Apparatus) and had resistances of 2.5–4 MΩ when filled with the following (in mM): 120 cesium methanesulfonate, 1 $MgCl_2$, 0.1 $CaCl_2$, 10 HEPES, 0.5 EGTA, 4 Mg-ATP, 0.3 Na-GTP (pH 7.2 with CsOH). The bath solution contained the following (in mM): 10 $BaCl_2$, 110 NaCl, 20 TEA-Cl, 5 4-AP, 10 HEPES, 2 $MgCl_2$, 3 KCl, 10 Glucose, 0.001 TTX (pH 7.4 with NaOH). Currents were recorded with an EPC 10 amplifier controlled by Patch Master Software (HEKA Elektronik Dr. Schulze GmbH, Germany). Linear leak and capacitive currents were digitally subtracted with a P/4 prepulse protocol. The current–voltage dependence was fitted according to a Boltzmann equation: $I=G_{max}\cdot(V-V_{rev}) / (1+exp((V-V1/2) / k))$ where G_{max} is the maximum conductance of endogenous calcium channels, V_{rev} is the extrapolated reversal potential of the calcium current, $V_{1/2}$ is the potential for half-maximal conductance, and k is the slope. For each test potential conductance was calculated as $G=I/(V-V_{rev})$, where the reversal potential (V_{rev}) was taken from the corresponding plotted I-V curve. Cells were depolarized from a holding potential of -70 mV to +60 mV with 10 mV steps, 10mM Barium was used as a charge carrier.

Recording of mEPSCs: Excitatory activity of neuronal cultures (spontaneous miniature postsynaptic currents, mEPSC) was recorded using the whole-cell patch-clamp technique at a holding potential of −70 mV. Patch pipettes were pulled from borosilicate glass (Harvard Apparatus), fire-polished (Microforge MF-830; Narishige), and had resistances of 2.5–4 MΩ when filled with the following (in mM): 130 Cs-methanesulfonate, 10 CsCl, 1 $MgCl_2$, 0.1 $CaCl_2$, 10 HEPES, 2 EGTA, 4 Mg-ATP, and 0.3 Na-GTP, pH 7.2 with KOH. The bath solution contained the following (in mM): 137 NaCl, 3 KCl, 10 HEPES, 2 $MgCl_2$, 1.8 $CaCl_2$, 10 glucose, 0.05 DL-AP5, 0.02 bicuculline and 0.0005 TTX, pH 7.4 with NaOH. Currents were recorded with an EPC 10 amplifier controlled by PatchMaster software (HEKA Elektronik Dr. Schulze GmbH, Germany) and analyzed using Mini analysis software (Symaptosoft, USA). Amplitude and frequency of mEPSC were normalized to the corresponding mean value of the heterozygote control condition for each experiment separately.

FM-dye loading. Live cell cultured hippocampal neuron at DIV 17-25 were pre-incubated in 2.5mM KCl Tyrode solution containing (130 mM NaCl, 2.5 mM KCl, 2 mM $CaCl_2$, 2 mM $MgCl_2*6H_2O$, 10 mM HEPES, 30 mM glucose, pH 7.4) in a specialized Ludin-chamber (Life Imaging services, CH-4057 Basel Switzerland) (18). To block network activity 10 µM CNQX and 50 µM AP5 (both Tocris Bioscience, Bristol, UK) was present in all solutions and the temperature was kept at 37°C. Cells were loaded with FM4-64 dyes upon 60 mM KCl depolarization followed by a continuous washout with Tyrode solution (2.5 mM) using an inverted Axiovert 200 M setup (Carl Zeiss Light Microscopy, Göttingen, Germany) connected to a Valve Link perfusion system. For quantification, FM4-64 and

eGFP images were matched. For analysis presynaptic varicosities of eGFP control and triple-knockout axons were selected that formed boutons along neighbouring non-transfected dendrites. Additionally, phase contrast images were taken in order to monitor overall cell morphology. Average fluorescent intensities of single boutons were quantified using Metavue software.

Calcium imaging. To determine presynaptic calcium influx, primary neurons were transfected at DIV6 with SynGCaMP6f (19) and co-transfected, as indicated with additional plasmids pHR-βA-mcherry-U6-shRNA-$\alpha_2\delta$-1, pHR-βA-mcherry, pβA-$\alpha_2\delta$-1. Nine to eleven days post transfection, coverslips were mounted in a recording chamber and placed on an inverted microscope (Olympus IX71, 60x, 1.42 NA PlanApo objective). The chamber was superfused at a rate of 1.0–1.5 ml/min with bath solution (temperature 32°C), containing (in mM): NaCl 145, KCl 3, MgCl2 1.5, CaCl2 2, glucose 11, HEPES 10; pH 7.3 adjusted with NaOH, to which to suppress postsynaptic signaling, 10 µM 6-cyano-7-nitroquinoxaline-2,3-dione (CNQX), 25 µM D, L-2-amino-5-phosphonovaleric acid (AP5), and 10 µM bicuculline were added. All chemicals were obtained from Sigma (St. Louis, MO, USA), except calcium channel blockers (Tocris). As described (17, 19) a stimulation electrode, custom built from two platinum wires of 10 mm length in 10 mm distance was positioned with a micromanipulator (MPC-200, Sutter Instrument, Novato, CA, USA) and neurons were stimulated with 50 Hz trains of 1, 3, and 10 current pulses (1 ms, 55 mA). Calcium transients were visualized and recorded (20 ms exposure time, frame rate 50 Hz, 200 frames, binning 2, pixel size: 0.215 µm per pixel) with a CMOS camera (Orca Flash4.0, Hamamatsu, Japan), using a halogen lamp light source (HXP 120) in the green channel (excitation: 470/40 nm, emission: 525/50 nm). Positive co-transfection with mcherry was determined and documented for the analysis in the red channel (excitation: 560/40 nm; emission: 630/75 nm). Recordings were controlled by Micromanager software (Vale Lab, UCSF). As a standard reference, 50 frames were recorded before the stimulus train was triggered used nm to calculate the fluorescent baseline. Recordings were analyzed in FIJI/ImageJ (National Institute of Health, MA, USA). 22 regions of interest (ROIs) per recorded cell were drawn around mcherry positive presynaptic boutons using the plugin 'Time Series Analyzer V3' with an AutoROI diameter of 10 pixels. The regions were subsequently used in the green SynGCaMP6f recordings to quantify the fluorescence changes. For this, the commonly used "Subtract Background…" tool of ImageJ (20) (employing a "rolling ball" algorithm with a radius of 20 pixels ≈ 4.3 µm) was applied to remove the background signal. The mean of the four highest fluorescence pixels was calculated for each ROI at each frame applying a self-made macro (19). To obtain subtraction pictures showing the regions with increase in the SynGCaMP6f-fluorescense (ΔF; Fig. 2A), the averaged picture of frame 30-49 (control) was subtracted from the average of 20 consecutive frames around the maximal response. Further analysis was done in Microsoft Excel, calculating for each ROI the changes in fluorescence as ΔF/F0 and averaging the 22 synapses per cell. Statistical analysis was done on the maxima of each cell in Graphpad Prism as indicated. For obtaining the cumulative frequency distributions, the maximal response (ΔF/F0) was calculated by averaging 5 frames of the peak signal for every single synapse in Microsoft Excel. For each condition and stimulation, we calculated a cumulative histogram with defined class sizes and normalized the values to the number of synapses per condition. Data are plotted as peak response (log) against the frequency (%) starting at 0.01 as signals below this threshold were indistinguishable from noise and hence considered as non-responding.

Immunocytochemistry. Immunolabeling of permeabilized neurons was performed as previously described (21). Briefly, neurons were fixed in pF (4% paraformaldehyde, 4% sucrose) in PBS at room temperature. Fixed neurons were incubated in 5% normal goat serum in PBS/BSA/Triton

(PBS containing 0.2% BSA and 0.2% Triton X-100) for 30 min. Primary antibodies were applied in PBS/BSA/Triton overnight at 4°C and detected by fluorochrome-conjugated secondary antibodies (Invitrogen). For staining of surface-expressed HA-tagged $\alpha_2\delta$ constructs, living neurons were incubated with the rat anti-HA antibody (1:100) for 30 min at 37 °C; coverslips were rinsed in HBSS and fixed in pF for 10 min. After fixation, neurons were washed with PBS for 30 min, blocked with 5% goat serum for 30 min, and labeled with anti-rat Alexa Fluor 594 (1:4000, 1h). Coverslips were mounted in p-phenylenediamine glycerol to retard photobleaching (22) and observed with an Axio Imager microscope (Carl Zeiss) using 63×, 1.4 NA oil-immersion objective lens or with an Olympus BX53 microscope (Olympus, Tokio, Japan) using a 60× 1.42 NA oil-immersion objective lens. Images were recorded with cooled CCD cameras (SPOT Imaging Solutions, Sterling Heights, MI USA and XM10, Olympus, Tokio, Japan).

Electron microscopy, structural analysis: Cultures of neurons were prepared as described above with the exception, that neurons were grown on coverglasses coated with a carbon layer as previously described (23), and fixed with 2% glutaraldehyde (Agar Scientific Ltd., Stansted, UK) in phosphate buffer (PB; 0.1 M, pH 7.4). After washing in PB three times 5 min at RT, neurons were post-fixed and stained with 0.2% osmium tetroxide (Electron Microscopy Sciences, Hatfield, PA) in PB (w/v) for 30 min at RT. After stopping reaction in PB, samples were washed in water four times 5 min at RT and stained with 0.25% uranyl-acetate (AL-Labortechnik e.U., Amstetten, Austria) in water (w/v) overnight at 4 °C. They were then dehydrated in graded ethanols, infiltrated with anhydrous acetone (Merck KGaA, Darmstadt, Germany), and embedded in Durcupan™ ACM resin (Fluka, Buchs, Switzerland) using propylene oxide (Sigma) as intermedium. For polymerization, BEEM capsules (Science Services, Munich, Germany) were filled with freshly prepared Durcupan™, inverted and placed onto the neuron cultures and cured for 48 h at 60 °C. Thereafter, coverslips were removed from the block surface with the neurons remaining in the block. Serial ultrathin sections (40 and 70 nm, respectively) were cut with an ultramicrotome UC7 (Leica Microsystems). Sections were collected onto Formvar-coated copper slot grids and stained with 1% aqueous uranyl acetate and 0.3% Reynold's lead citrate. They were examined in a Philips Tecnai 10 transmission electron microscope (TEM; Thermo Fisher Scientific GmbH) at 80 kV, equipped with a side-mounted camera MegaView III G3 (Electron Microscopy Soft Imaging Solutions [EMSIS] GmbH; Muenster, Germany). Images were processed with Radius software (EMSIS) and Photoshop (Adobe®) without changing any specific feature.

Pre-embedding immunoelectron microscopy: Cultures of neurons were prepared as described above and fixed with 4% formaldehyde, 0.05% glutaraldehyde and 15% of a saturated picric acid solution (Sigma) in PB for 10 minutes at room temperature. To increase penetration of reagents, fixed neurons were infiltrated with increasing gradients of sucrose (5, 10 and 20%) in PB (w/v) 1h each at 4°C, flash-frozen on liquid nitrogen and rapidly thawed in lukewarm PB. Cells were then washed in Tris-buffered saline (TBS; 0.05 M, 0.9% NaCl, pH 7.4) and incubated in 10% normal goat serum (v/v) plus 2% BSA (w/v) in TBS for 1h at RT for blocking of nonspecific binding sites. Rabbit anti-GFP primary antibodies (Abcam, Cambridge, UK) were then applied in TBS plus 2% BSA at a concentration of 0.125 µg/ml overnight at 4 °C. Cells were washed four times 5 min at RT and incubated with 1.4 nm nanogold conjugated secondary antibodies (Nanoprobes, Yaphank, NY) in TBS plus 2% BSA at a concentration of 0.4 µg/ml overnight at 4 °C. Cells were washed in TBS four times 5 min at RT and post-fixed with 1% glutaraldehyde in TBS for 20 min at RT. After thorough wash in water, gold particles were silver-amplified using an HQ Silver™ enhancement kit (Nanoprobes). Samples were then processed directly for Durcupan™ embedding as described

above. Alternatively, fixed neurons were treated with 0.1% Triton X-100 in TBS (T-TBS) for 20 min at RT for increasing penetration of reagents instead of the freeze-thaw procedure described above. Consequently, primary antibodies and nanogold-conjugated secondary antibodies were also diluted in T-TBS. All other conditions as antibody concentrations, infiltration times and washing steps stood the same.

Antibodies. Primary antibodies were used as follows: rb-polyclonal anti-synapsin (1:20.000, 1:500 in combination with A350), m-monoclonal anti-synapsin-1, clone 46.1 (1:2.000, 1:500 in combination with A350), rb-polyclonal anti-$Ca_v2.1$ (1:2.000), rb-polyclonal anti-$Ca_v2.2$ (1:2.000), rb-polyclonal anti-vGAT (1:2.000, 1:500 in combination with A350), rb-polyclonal anti vGLUT1 (1:20.000) all from Synaptic Systems (Göttingen, Germany). Further antibodies were m-monoclonal anti-PSD-95 (1:1.000, Affinity Bioreagent, Golden CO, USA), rat-monoclonal-anti-HA (1:1.000, Roche Diagnostics, Vienna, Austria), m-monoclonal anti $GABA_AR$ (1:500, Chemicon/EMD Millipore, Billerica, MA, USA) and rb-polyclonal anti-GFP (1:4000, Abcam ab6556, Cambridge, UK). Secondary antibodies used were as follows: goat anti-mouse Alexa 594 (1:4.000); goat anti-rabbit Alexa 350 (1:500), and Alexa 594 (1:4.000), and goat anti-rat Alexa 594 (1:4.000), all from Invitrogen (Fisher Scientific, Vienna, Austria); Fab' fragments of goat anti-rabbit IgG conjugated to 1.4-nm gold particles (1:200, Nanoprobes, Yaphank, NY).

Analysis and quantification.

Surface and synaptic expression of $\alpha_2\delta$ isoforms: In order to analyze the detailed synaptic localization of individual $\alpha_2\delta$ isoforms, each subunit was N-terminally tagged with a 2HA tag as described. A rat anti HA antibody (Invitrogen) was used to detect 2HA-tagged subunits. To quantify the distribution of eGFP, synapsin (A350) as well as 2HA-$\alpha_2\delta$-1/-2/-3 (anti-HA/A594) signals, average fluorescent intensities were measured along a line through the respective synaptic bouton. Relative fluorescent intensities were processed in MS excel and finally illustrated with Photoshop CS6. To analyze surfaxe expression of anti-HA-labeled $\alpha_2\delta$ subunits, a threshold was set for each image in the anti-rat-HA (A594) channel and fluorescent intensity measurements were performed as follows. Axons have been distinguished from dendrites by morphological criteria based on the fluorescence of soluble eGFP. Hence, axons are evident as smooth processes with a constant diameter over millimeters which, whenever contacting neighboring neurons, form presynaptic boutons. In contrast, the dendrites taper within ~150 to 300 μm from the soma and are covered by numerous dendritic spines throughout (e.g. (6, 21)). To measure the axon hillock, fluorescent intensity of the anti-HA/$\alpha_2\delta$ signal for each individual HA-tagged isoform was recorded along a line region of 20μm extending from the soma into the axon using Metamorph software. Axon intensity was measured in the proximal axon along a 30μm line region adjacent to the axon hillock. For dendritic surface expression the anti-HA fluorescent signal was quantified over a region of 30μm on each of three different primary dendrites per neuron. Measurements were background subtracted and averaged for each cell and region prior to statistical analysis.

Synaptic co-localization: In order to visualize the detailed synaptic localization of each presynaptic (Syn, $Ca_V2.1$, $Ca_V2.2$, vGLUT1, vGAT) as well as postsynaptic marker (PSD-95, $GABA_A$-R, AMPA-R) in control, triple-knockout and $\alpha_2\delta$-rescue conditions, linescan analysis was performed. To this end the distribution of the eGFP (A488) signal, the synapsin (A350) as well as the $Ca_V2.1$, $Ca_V2.2$ or PSD-95 (A594) signal, was measured along a line of 3μm cutting the respective synaptic bouton similar to the localization experiments. Average fluorescent intensities were background subtracted, plotted in MS excel and finally illustrated with Photoshop CS6.

Single bouton quantification. Regions with spreading axons from hippocampal neurons (DIV 18-22) were selected in the eGFP channel.

To select presynaptic synapses or axonal varicosities (putative synaptic boutons), cover glasses were systematically screened for contact sites between axons of presynaptic neurons (transfected with the fluorescent markers eGFP or mCherry and dendrites or somata of nontransfected postsynaptic neurons. Images of randomly selected well differentiated cells were acquired with the same exposure and gain settings for all conditions within an experiment. These settings were initially adjusted to facilitate optimal visualization of peripheral cell compartments (axons and presynaptic boutons) also in weakly expressing neurons. Therefore, overly saturated neurons (based on eGFP and mCherry levels) were excluded from analysis and only cells with medium to low eGFP or mCherry expression were selected for further analysis. eGFP positive varicosities (putative synapses) were selected and the threshold was set in order to cover the entire area of individual boutons. By using the shrink region to fit tool (Metavue), each putative synapse was further measured for colocalization with synapsin (A350) or the $Ca_V2.1$ (A594) fluorescent signal in the respective corresponding micrographs. In each channel-micrograph a separate background region was selected and subtracted from the average fluorescent intensities. For $Ca_V2.2$, PSD-95, $GABA_A$-R and AMPA-R quantification a slightly modified protocol was used because these staining patterns were, due to their subsynaptic localization, not directly co-localizing with the presynaptic corresponding eGFP signal. Therefore, the presynaptic ROI was dilated by 0.5 µm in order to avoid false positive or false negative staining patterns. In each custom-built macro (journal) it was therefore possible to measure the presynaptic marker synapsin together with $Ca_V2.1$/$Ca_V2.2$, PSD-95, $GABA_A$-R and AMPA-R in a blinded manner. The following parameters were selected for quantification: Average fluorescent intensity/integrated fluorescent intensity/relative area of the bouton. For each neuron an average of 40-50 presynaptic varicosities were analyzed in 3-5 independent culture preparations for each condition. Single bouton quantification of GABAergic MSNs co-cultured with glutamatergic cortical neurons was done from two independent culture preparations for each condition as described (9). Further data analysis was performed with MS Excel and Graph Pad Prism. In the presented micrographs of eGFP-filled axonal varicosities/synaptic boutons black-level was adjusted to avoid strong over-saturation of the more-intensely labeled boutons. Consequently, the thin axonal processes are not always clearly visible in the presented eGFP images. Therefore, a dotted line was used to outline the eGFP-filled axon and bouton using the free transform pen tool of Photoshop CS6. Grayscale images of the eGFP bouton and axon served as a template to draw the outlines, whereby the black-levels were temporarily adjusted by linearly shifting the graduation curves to visualize the entire axonal segment and bouton. Lines were then transferred to the corresponding images of co-labeled antibodies (e.g. $Ca_V2.1$, synapsin) in order to better visualize the position of the synaptic clusters in relation to the eGFP-filled axonal bouton in the different channels. In the final presented micrographs black-levels for each channel were adjusted separately by linearly shifting the graduation curves in Photoshop CS6 in order to minimize background and maximize the signal intensity.

Electron microscopy: Sampling areas were chosen at random from different regions of the thin sections for each sample per neuron culture and condition. Perpendicular cut excitatory spine synapses were randomly selected and micro-graphed within these areas. The length of presynaptic active zone and PSD was then measured for each synapse using ImageJ software (40 and 50 synapses each for unlabeled control and immunolabelled cultures, respectively) according to the

following criteria: The presynaptic active zone was identified by i) an increased electron density of the synaptic membrane emerging significantly thickened in electron micrographs, ii) dense projections extending into the cytoplasm of the synaptic bouton, iii) rigid alignment of the presynaptic and postsynaptic membranes, and iv) a high electron density in the synaptic cleft. On the other hand, PSDs were identified by fuzzy electron-dense material extending from the plasma membrane into the cytoplasm of postsynaptic spines. For quantifying the PSD extension, ten measurements were randomly made per synapse including the minimum and maximum extension, and the mean value for each synapse was calculated (n=50 for each condition).

Statistical analysis. Results are expressed as means ± S.E. except where otherwise indicated, the type of statistical test used is given in the respective figure legends. The statistical requirements and assumptions underlying each test were evaluated for each data set (e.g. data normality, independence, similarity of variances, etc.) and, if violated, and alternative non-parametric test was selected for the analysis as indicated. Data were organized and analyzed using MS Excel and Graph Pad Prism (Graph Pad Software, La Jolla, CA, USA). Graphs and figures were generated using Graph Pad Software, Adobe Photoshop CS6, and Affinity Photo.

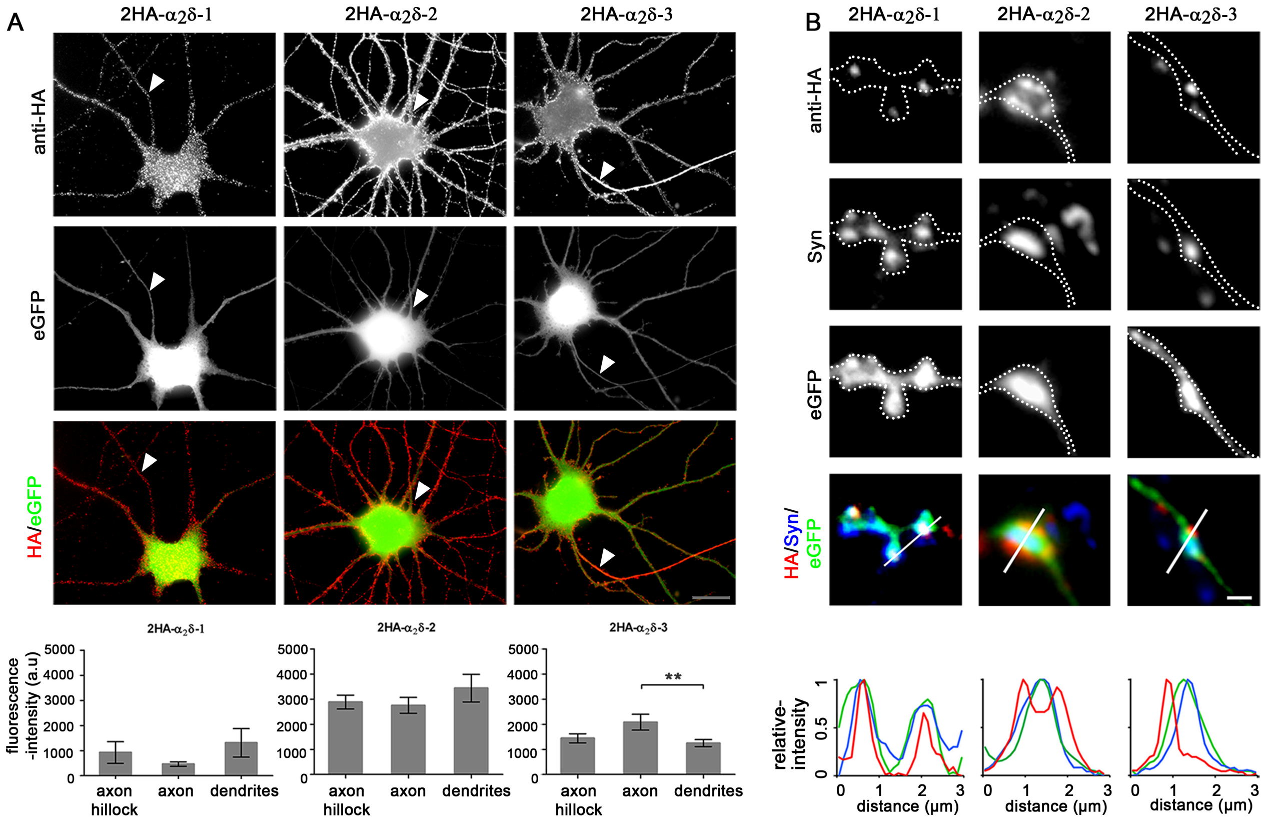

Fig. S1. Subcellular distribution of the three neuronal $\alpha_2\delta$ subunits. Cultured hippocampal neurons were transfected with 2HA-tagged $\alpha_2\delta$ subunits together with soluble eGFP to outline the neuronal morphology and live cell labeled for the HA epitope. (A) Live cell staining revealed a strong expression of all three $\alpha_2\delta$ isoforms on the soma, dendrites, and axons (arrowheads). Overall, $\alpha_2\delta$-2 surface expression was up to two-fold higher when compared to $\alpha_2\delta$-1 or $\alpha_2\delta$-3, respectively, and $\alpha_2\delta$-3 specifically accumulated in the axon. To visualize the comparatively low expression of $\alpha_2\delta$-1 on the cell soma, the selected image is focused on the somatic cell surface. Average fluorescence intensity measurements are shown for axon hillocks, axons, and dendrites. Statistics were performed with one-way ANOVA, followed by Holm-Sidak post-hoc test; $\alpha_2\delta$-1: $F_{(2,9)}=0.02$, $p=0.99$; $\alpha_2\delta$-2: $F_{(2,12)}=0.83$, $p=0.46$; $\alpha_2\delta$-3: $F_{(2,35)}=4.0$, $p=0.027$; $^{**}p=0.01$; data from 3 culture preparations, error bars indicate SEM. Scale bar, 20µm. (B) All $\alpha_2\delta$ isoforms showed a synaptic localization, which is supported by the overlay or juxtaposition of the linescan peaks of synapsin (blue), HA-$\alpha_2\delta$ (red), and eGFP (green). $\alpha_2\delta$-2 specifically accumulated in the perisynaptic membrane around the central synapsin label. Scale bar, 1µm.

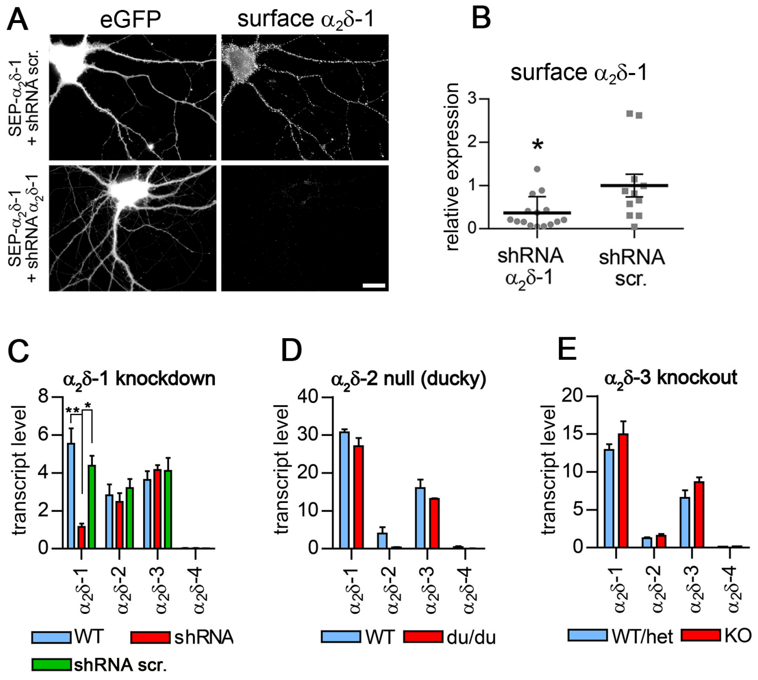

Fig. S2. shRNA knockdown of α₂δ-1 protein and mRNA in cultured hippocampal neurons. (A) Live cell surface staining of neurons expressing α₂δ-1 with an extracellular super-ecliptic pHluorin tag (SEP-α₂δ-1) reveals robust knockdown of protein expression by α₂δ-1 specific shRNA (lower panel) when compared to scrambled control shRNA. Scale bar, 20 µm. (B) shRNA decreased surface expression of SEP-α₂δ-1 to 37±10% (mean±SD) of control neurons transfected with scrambled shRNA [$t_{(12)}$=2.3, p=0.044]. (C-E) qPCR expression profiles of the four α₂δ isoforms in three different α₂δ deficient model systems. (C) Lentiviral transfection of cultured hippocampal neurons (DIV 24; ~90% transfection efficiency) with α₂δ-1 specific shRNA (shRNA; red bars) significantly reduced α₂δ-1 transcript levels to 25±3% (p=0.015) of neurons transfected with scrambled control shRNA (green bars) and to 21±6% (p=0.005) of untransfected neurons (WT; blue bars; ANOVA with Holm-Sidak posthoc test). Loss of either α₂δ-2 in ducky mice (D, α₂δ-2 null) or α₂δ-3 (E, α₂δ-3 knockout mice) did not induce compensational changes in the expression levels of the other isoforms. Mutated α₂δ-2 mRNA in ducky mice is unstable and thus strongly reduced, whereas due to the design of the α₂δ-3 knockout construct (introduction of a frameshift in exon 15) and the position of the α₂δ-3-specific Taqman assay (junction exon 5 and 6) the α₂δ-3 mRNA with the Lac-Z insert is stably expressed. [n-numbers: α₂δ-1, 3 culture preparations; ducky, 2 hippocampus preparations from 8-week-old mice; α₂δ-3 knockout, 3 hippocampus preparations from 8-week-old mice]

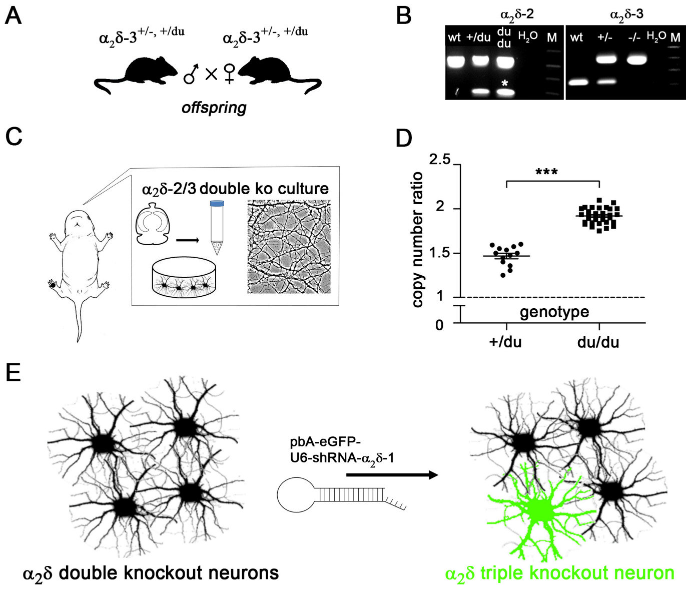

Fig. S3. Establishing a cellular α₂δ subunit triple knockout/knockdown model. (A) Crossbreeding double heterozygote $\alpha_2\delta$-2$^{+/du}$ / $\alpha_2\delta$-3$^{+/-}$ mice yielded $\alpha_2\delta$-2/-3 double knockout pups at an actual occurrence of ~3% (n=9 double knockout of 260 pups) compared to the expected Mendelian probability of 6.25%. (B) On P0 neonatal pups were individually marked by paw-tattooing. The $\alpha_2\delta$-2 wildtype allele was identified by a 541 bp band (wt). The presence of the du allele in heterozygote (+/du) and homozygote ducky (du/du) mice was indicated by an additional band at ~280 bp (see Materials and Methods for details), whereby homozygosity (du/du) was suggested by an increased intensity of the ~280 bp band (asterisk). Genotyping for $\alpha_2\delta$-3 revealed bands at 183 bp for wt, 331 and 183 bp for heterozygote, and 331 bp for homozygote knockout mice. (C) Putative double knockout pups ($\alpha_2\delta$-3$^{-/-,}$ $\alpha_2\delta$-2$^{du/du}$) were selected for hippocampal culture preparation in parallel with control littermates. (D) Due to the large genomic rearrangement in ducky mice, the ducky mutation required a final confirmation employing a copy number counting qPCR approach. The graph indicates experimentally determined copy number ratios from 32 (wt and du/du) and 13 (+/du) mice: wt ($\alpha_2\delta$-2$^{+/+}$), 2 copies normalized to a ratio of 1 (dashed line); heterozygote ($\alpha_2\delta$-2$^{+/du}$), 3 copies a ratio of ~ 1.5; homozygote ($\alpha_2\delta$-2$^{du/du}$), 4 copies a ratio of ~2.0. (ANOVA on ranks with Dunn's post-hoc test, ***$p<0.001$). (E) Ultimately, in confirmed $\alpha_2\delta$-2/-3 double knockout cultured hippocampal neurons $\alpha_2\delta$ triple knockout neurons were established by transfection with $\alpha_2\delta$-1 shRNA (light green).

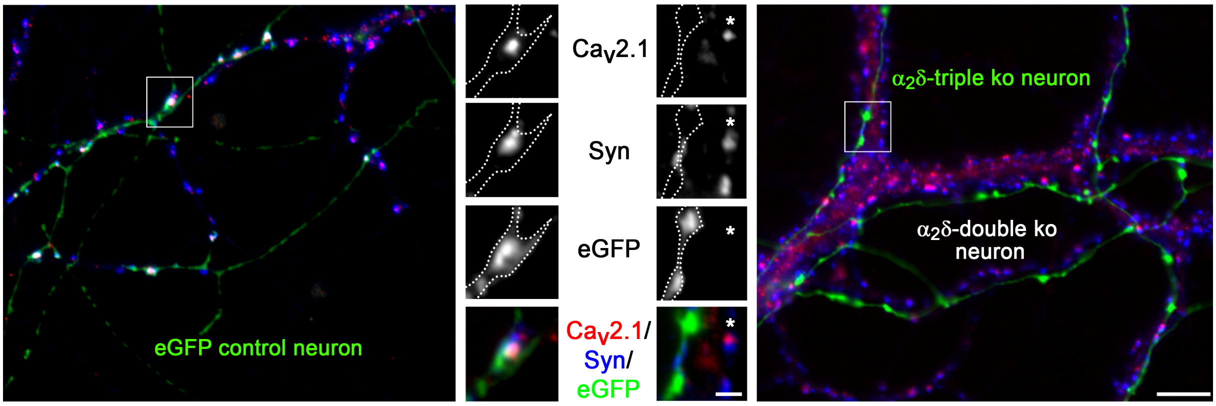

Fig. S4. Axons from $\alpha_2\delta$ subunit triple knockout neurons form varicosities along postsynaptic neurons. Left panel: In cultured hippocampal neurons axonal processes can be observed as thin smooth processes identified by the soluble eGFP fluorescence with a constant diameter which can be followed over millimeters. Whenever these processes are contacting cell somata or dendrites of neighboring neurons they form varicosities which, based on the expression of presynaptic calcium channels (Cav2.1) and synapsin (Syn), can be identified as presynaptic boutons (see magnified selection). Right panel: Axons from eGFP positive triple knockout neurons displayed varicosities similar to control neurons. These varicosities are typically observed in the vicinity of dendrites or somata of non-transfected neighboring cells. However, the majority of these varicosities lacked staining for synapsin and $Ca_V2.1$ (see magnified selection). Importantly, in this experimental paradigm axons and varicosities from triple knockout neurons can be directly compared to eGFP negative double knockout synapses (innervating the same postsynaptic dendrites, red/$Ca_V2.1$, blue/synapsin; asterisks in the magnified selection). Scale bars, 1µm and 5µm.

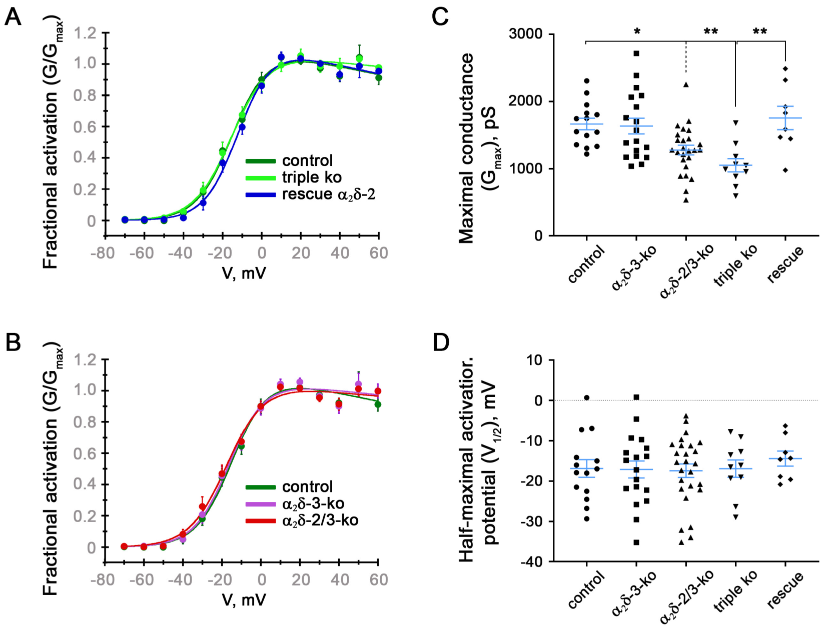

Fig. S5. α₂δ subunit triple knockout reduces conductance of total somato-dendritic calcium channel but does not affect the voltage-dependence of activation. Fractional activation (A, B) of the total calcium channel population and half-maximal activation potential (D) remains unchanged upon deletion of one (α₂δ-3), two (α₂δ-2/3), or all (triple ko) α₂δ subunits. (C) Maximal conductance is reduced in triple knockout (triple ko) and double knockout (α₂δ-2/3-ko) when compared with control or α₂δ-2 rescued (rescue) neurons. (ANOVA: $F_{(4,70)}$=6.8, p<0.001; Tukey post-hoc test: *p<0.05, **p<0.01; 8-26 cells from 5 culture preparations; Horizontal lines represent means, error bars ±SEM.)

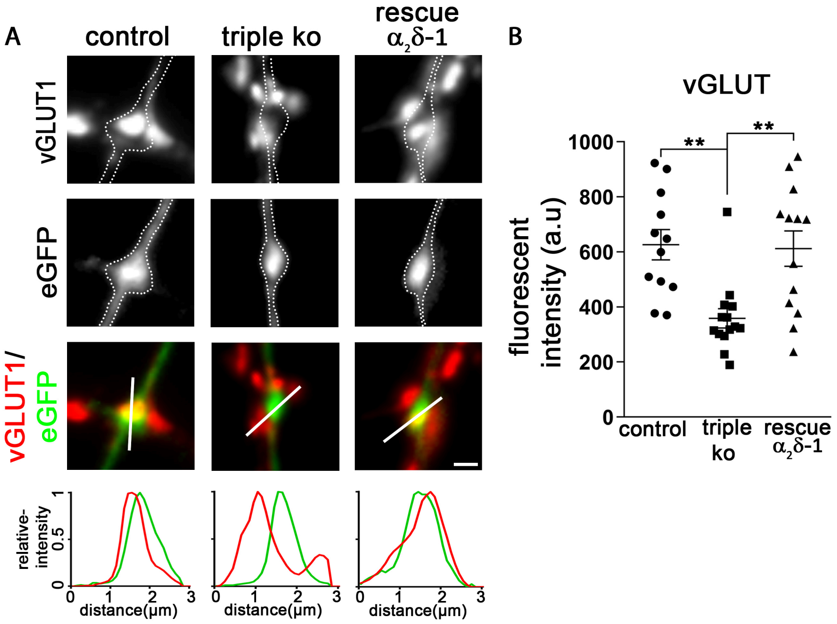

Fig. S6 Failure of presynaptic vGLUT1 clustering in α₂δ triple knockout boutons. (A) Immunofluoresence analysis of axonal varicosities from wildtype/heterozygote littermates (control, neurons transfected with eGFP only), triple knockout neurons (triple ko, $\alpha_2\delta$-2/-3 double knockout neurons transfected with shRNA-$\alpha_2\delta$-1 plus eGFP), and triple knockout neurons expressing $\alpha_2\delta$-1 (rescue, α2δ-2/-3 double knockout neurons transfected with shRNA-$\alpha_2\delta$-1 plus eGFP and $\alpha_2\delta$-1). Putative presynaptic boutons were identified as eGFP-filled axonal varicosities along dendrites of untransfected neurons (confer Fig. S4) and outlined with a dashed line. Outlines indicate synaptic boutons from control, triple knockout, as well as $\alpha_2\delta$-1 rescued neurons. The failure in synapse formation is indicated by the highly reduced intensity of vGLUT1 in the triple knockout condition, while neighboring eGFP-negative $\alpha_2\delta$-2/-3 double knockout boutons still show vGLUT1 clustering (asterisks). (B) Quantification of the fluorescent intensities of individual synaptic boutons from control, triple ko, and α2δ-1 rescued neurons. (ANOVA: $F_{(2,36)}=8.7$, $p<0.001$; Tukey post-hoc test: **$p<0.01$; 300-450 synapses in 10-14 individual cells from 2 culture preparations). Scale bar, 1µm.

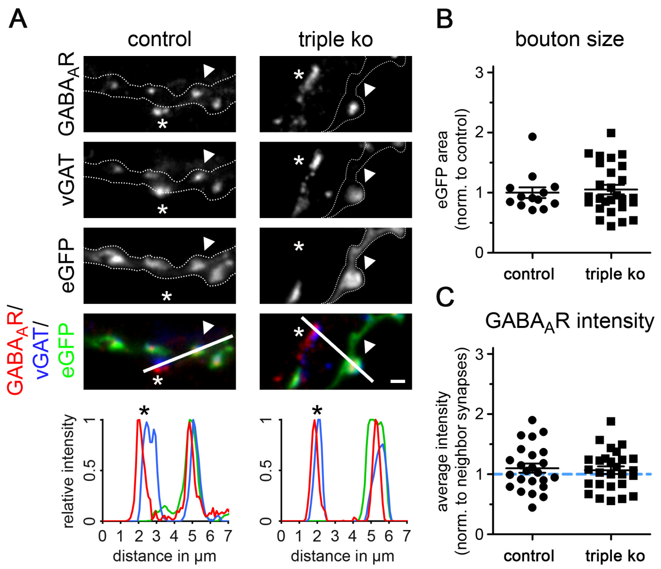

Fig. S7. GABAergic synapses can form in the absence of α2δ subunits. (A) Exemplary immunofluoresence micrographs of GABAergic MSNs immunolabeled for vGAT and the GABA$_A$R (22-24 DIV). Postsynaptic GABA$_A$R and presynaptic vGAT immunoreactivities were present in transfected α2δ-3 knockout (control) and triple knockout (triple ko) MSNs when compared to untransfected neighboring synapses (asterisks), resulting in co-localized fluorescence signals (individual linescans). Untransfected neighboring synapses were either knockout for α2δ-3 (in control condition) or α2δ-2/-3 double knockout (in triple ko condition). (B, C) Quantification of bouton size (B) and average GABA$_A$R fluorescence intensities (C). Values were normalized to eGFP area in control (B) or endogenous GABA$_A$R intensities of untransfected GABAergic neighboring synapses (C, indicated with a dashed line) within each culture preparation. (t-test: bouton size, $t_{(38)}$=0.4, p=0.69, 13-27 cells from 3 culture preparations; GABA$_A$R: $t_{(49)}$=0.3, p=0.73, 24-27 cells from 4 culture preparations). Values for individual cells (dots) and means (lines) ± SEM are shown. Scale bar, 1 µm.

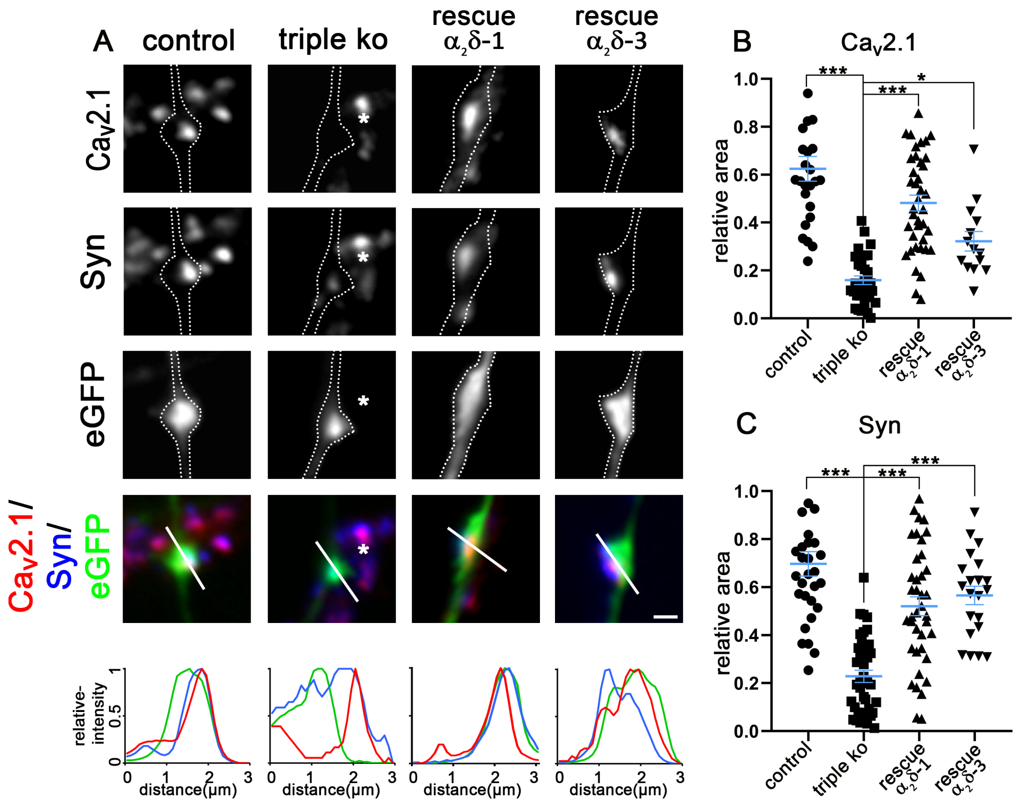

Fig. S8. Over-expression of $\alpha_2\delta$-1 and $\alpha_2\delta$-3 subunits rescues presynaptic $Ca_V2.1$ and synapsin clustering in triple knockout neurons. (A) Immunofluoresence analysis of axonal varicosities from wildtype neurons (control, neurons transfected with eGFP only), triple knockout neurons (triple ko, $\alpha_2\delta$-2/-3 double knockout neurons transfected with shRNA-$\alpha_2\delta$-1 plus eGFP), triple knockout neurons expressing $\alpha_2\delta$-1 (rescue, $\alpha_2\delta$-2/-3 double knockout neurons transfected with shRNA-$\alpha_2\delta$-1 plus eGFP and $\alpha_2\delta$-1), and triple knockout neurons expressing $\alpha_2\delta$-3 (rescue, $\alpha_2\delta$-2/-3 double knockout neurons transfected with shRNA-$\alpha_2\delta$-1 plus eGFP and $\alpha_2\delta$-3). Putative presynaptic boutons were identified as eGFP-filled axonal varicosities along dendrites of untransfected neurons (confer Suppl. Fig. 4) and outlined with a dashed line. Outlines indicate synaptic boutons from control, triple ko as well as $\alpha_2\delta$-3 rescued neurons. The failure in synapse formation is indicated by the highly reduced area of synapsin as well as presynaptic $Ca_V2.1$. Interestingly this defect could be rescued by each individual $\alpha_2\delta$-isoform (shown are $\alpha_2\delta$-1 and $\alpha_2\delta$-3, see text for $\alpha_2\delta$-2). The redundant function of $\alpha_2\delta$ subunits in synapse formation and calcium channel targeting is further supported by the fact that neighboring eGFP-negative $\alpha_2\delta$-2/-3 double ko boutons still containing $\alpha_2\delta$-1 formed synapses together with proper $Ca_V2.1$ abundance (asterisks). (B, C) Quantification of the fluorescence intensities of presynaptic $Ca_V2.1$ and synapsin clustering in control, triple knockout, and $\alpha_2\delta$-1 or $\alpha_2\delta$-3-expressing (rescued) triple knockout neurons. (ANOVA: $Ca_V2.1$, $F_{(3,107)}=33$, $p<0.001$; Synapsin, $F_{(3,125)}=28$, $p<0.001$; Tukey post-hoc test, *$p<0.05$, ***$p<0.001$; 400-1560 synapses from 10-39 cells from 2-5 culture preparations). Values for individual cells (dots) and means (lines) ± SEM are shown. Scale bar, 1 µm.

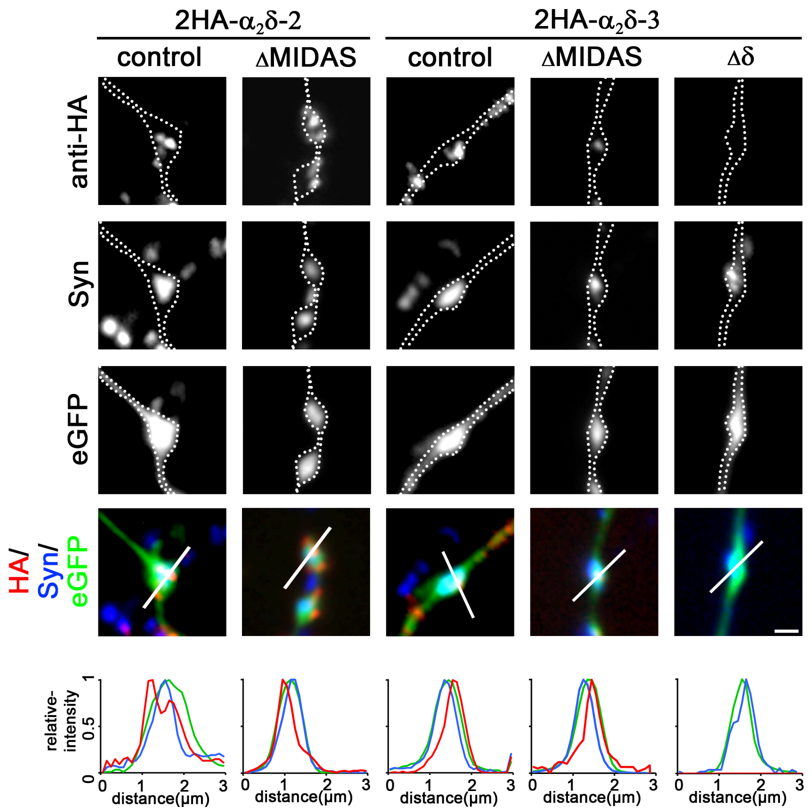

Fig. S9. HA-tagged $\alpha_2\delta$-ΔMIDAS constructs are expressed at the presynaptic membrane. Live-cell surface labeling (anti-HA) of 2HA-$\alpha_2\delta$-2 and 2HA-$\alpha_2\delta$-3 constructs without (control) or with a mutated MIDAS site (ΔMIDAS). Synapsin clustering (Syn) within eGFP-filled axonal varicosities confirm the presynaptic identity of the boutons. Both, 2HA-$\alpha_2\delta$-2-ΔMIDAS and 2HA-$\alpha_2\delta$-3-ΔMIDAS show a surface expression in presynaptic boutons similar to 2HA-$\alpha_2\delta$-2 and 2HA-$\alpha_2\delta$-3. The 2HA-α_2-3-Δδ (Δδ) construct, which lacks the membrane-anchor-containing δ subunit, does not show presynaptic membrane expression and hence serves as a negative control. Line scans visualize the synaptic localization within individual eGFP-positive synaptic boutons. Exemplary selections from 20 neurons (~600 imaged synapses) from 2-3 culture preparations. Scale bar, 1µm.

Table S1. Effect of $\alpha_2\delta$ knockout on current properties of total endogenous calcium channels.

	control			triple ko			rescue $\alpha_2\delta$-2		
	Mean	±SEM	n	Mean	±SEM	n	Mean	±SEM	n
CD (pA/pF)	-75.3	5.8	14	-32	3.6	10	-71.3	6.6	8
$V_{1/2}$(mV)	-16.9	2.2	14	-16.9	2.2	10	-14.4	1.9	8
V_{rev} (mV)	48.4	0.6	14	34.6	2.4	10	46.4	1.7	8
G_{max} (nS)	1.7	0.1	14	1.1	0.1	10	1.7	0.2	8

CD, current density; $V_{1/2}$, half-maximal activation potential; V_{rev}, reversal potential, G_{max}, maximum conductance

	control			$\alpha_2\delta$-3 ko			$\alpha_2\delta$-2/3 ko		
	Mean	±SEM	n	Mean	±SEM	n	Mean	±SEM	n
CD (pA/pF)	-75.3	5.8	14	-66.3	4.2	18	-51.6	3.5	26
$V_{1/2}$(mV)	-16.9	2.2	14	-17.2	2.1	18	-17.4	1.8	26
V_{rev} (mV)	48.4	0.6	14	46.7	1.0	18	44.7	1.4	26
G_{max} (nS)	1.7	0.1	14	1.6	0.1	18	1.3	0.1	26

CD, current density; $V_{1/2}$, half-maximal activation potential; V_{rev}, reversal potential, G_{max}, maximum conductance

Movie S1. Presynaptic calcium signals in response to 1, 3, or 10 APs in double-heterozygous control neurons. Representative recordings of synGCaMP6f fluorescence from axonal varicosities of control neurons (double-heterozygous) in response to 1, 3, and 10 AP stimulations. Recordings were acquired using a 60x 1.42 NA oil objective and an Orca Flash4.0 CMOS camera (Hamamatus Photonics) at a frame rate of 50Hz (binning 2, pixel size: 0.215 µm). Image stacks were converted to avi files in ImageJ or Metamorph and the movie was arranged in Camtasia (TechSmith). In the movie the recordings are played at original speed (first video sequence, speed 1x) and slowed down 4-fold (second video sequence, speed 0.25x) for better recognition of the fluorescent signals. Stimulations were triggered after 1 second, as indicated by the appearance of a yellow dot. The increasing fluorescent signals of single synapses in response to stimulation by an increasing number of APs can be clearly recognized.

Movie S2. Presynaptic calcium signals in response to 1, 3, or 10 APs in triple knockout neurons. Representative recordings of synGCaMP6f fluorescence from axonal varicosities of control neurons (double-heterozygous) in response to 1, 3, and 10 AP stimulations. Recordings were acquired using a 60x 1.42 NA oil objective and an Orca Flash4.0 CMOS camera (Hamamatus Photonics) at a frame rate of 50Hz (binning 2, pixel size: 0.215 µm). Image stacks were converted to avi files in ImageJ or Metamorph and the movie was arranged in Camtasia (TechSmith). In the movie the recordings are played at original speed (first video sequence, speed 1x) and slowed down 4-fold (second video sequence, speed 0.25x) for better recognition of the fluorescent signals. Stimulations were triggered after 1 second, as indicated by the appearance of a yellow dot. While in control neurons the changes in fluorescence in response to stimulation by an increasing number of APs can be recognized (movie S1), in triple knockout neurons the fluorescence signals particularly in response to 1AP, but also to 3AP, are hardly detectable.

SI References

1. J. Brodbeck *et al.*, The ducky mutation in Cacna2d2 results in altered Purkinje cell morphology and is associated with the expression of a truncated alpha 2 delta-2 protein with abnormal function. *J Biol Chem* **277**, 7684-7693 (2002).
2. T. D. Schmittgen, K. J. Livak, Analyzing real-time PCR data by the comparative C(T) method. *Nat Protoc* **3**, 1101-1108 (2008).
3. B. Schlick, B. E. Flucher, G. J. Obermair, Voltage-activated calcium channel expression profiles in mouse brain and cultured hippocampal neurons. *Neuroscience* **167**, 786-798 (2010).
4. V. Di Biase *et al.*, Surface traffic of dendritic CaV1.2 calcium channels in hippocampal neurons. *J Neurosci* **31**, 13682-13694 (2011).
5. G. J. Obermair, W. A. Kaufmann, H. G. Knaus, B. E. Flucher, The small conductance Ca2+-activated K+ channel SK3 is localized in nerve terminals of excitatory synapses of cultured mouse hippocampal neurons. *Eur J Neurosci* **17**, 721-731 (2003).
6. G. J. Obermair, Z. Szabo, E. Bourinet, B. E. Flucher, Differential targeting of the L-type Ca2+ channel alpha 1C (CaV1.2) to synaptic and extrasynaptic compartments in hippocampal neurons. *Eur J Neurosci* **19**, 2109-2122 (2004).
7. S. Kaech, G. Banker, Culturing hippocampal neurons. *Nat Protoc* **1**, 2406-2415 (2006).
8. R. I. Stanika, I. Villanueva, G. Kazanina, S. B. Andrews, N. B. Pivovarova, Comparative impact of voltage-gated calcium channels and NMDA receptors on mitochondria-mediated neuronal injury. *J Neurosci* **32**, 6642-6650 (2012).
9. S. Geisler *et al.*, Presynaptic alpha2delta-2 Calcium Channel Subunits Regulate Postsynaptic GABAA Receptor Abundance and Axonal Wiring. *J Neurosci* **39**, 2581-2605 (2019).
10. G. J. Obermair *et al.*, The Ca2+ channel alpha2delta-1 subunit determines Ca2+ current kinetics in skeletal muscle but not targeting of alpha1S or excitation-contraction coupling. *J Biol Chem* **280**, 2229-2237 (2005).
11. M. Fischer, S. Kaech, D. Knutti, A. Matus, Rapid actin-based plasticity in dendritic spines. *Neuron* **20**, 847-854 (1998).
12. A. Bikbaev *et al.*, Auxiliary α2δ1 and α2δ3 Subunits of Calcium Channels Drive Excitatory and Inhibitory Neuronal Network Development. *J Neurosci* **40**, 4824-4841 (2020).
13. T. N. Petersen, S. Brunak, G. von Heijne, H. Nielsen, SignalP 4.0: discriminating signal peptides from transmembrane regions. *Nat Methods* **8**, 785-786 (2011).
14. S. B. Ellis *et al.*, Sequence and expression of mRNAs encoding the alpha 1 and alpha 2 subunits of a DHP-sensitive calcium channel. *Science* **241**, 1661-1664 (1988).
15. N. Qin, S. Yagel, M. L. Momplaisir, E. E. Codd, M. R. D'Andrea, Molecular cloning and characterization of the human voltage-gated calcium channel alpha(2)delta-4 subunit. *Mol Pharmacol* **62**, 485-496 (2002).
16. P. Subramanyam *et al.*, Activity and calcium regulate nuclear targeting of the calcium channel beta4b subunit in nerve and muscle cells. *Channels (Austin)* **3**, 343-355 (2009).
17. J. Brockhaus *et al.*, α-Neurexins Together with α2δ-1 Auxiliary Subunits Regulate Ca2+ Influx through Cav2.1 Channels. *The Journal of Neuroscience* **38**, 8277-8294 (2018).
18. B. Nimmervoll, B. E. Flucher, G. J. Obermair, Dominance of P/Q-type calcium channels in depolarization-induced presynaptic FM dye release in cultured hippocampal neurons. *Neuroscience* **253**, 330-340 (2013).
19. J. Brockhaus, B. Bruggen, M. Missler, Imaging and Analysis of Presynaptic Calcium Influx in Cultured Neurons Using synGCaMP6f. *Front Synaptic Neurosci* **11**, 12 (2019).
20. S. Iwabuchi, Y. Kakazu, J. Y. Koh, N. C. Harata, Evaluation of the effectiveness of Gaussian filtering in distinguishing punctate synaptic signals from background noise during image analysis. *J Neurosci Methods* **223**, 92-113 (2014).
21. G. J. Obermair *et al.*, Reciprocal interactions regulate targeting of calcium channel beta subunits and membrane expression of alpha1 subunits in cultured hippocampal neurons. *J Biol Chem* **285**, 5776-5791 (2010).

22. B. E. Flucher *et al.*, Triad formation: organization and function of the sarcoplasmic reticulum calcium release channel and triadin in normal and dysgenic muscle in vitro. *J Cell Biol* **123**, 1161-1174 (1993).
23. M. Campiglio *et al.*, STAC proteins associate to the IQ domain of CaV1.2 and inhibit calcium-dependent inactivation. *Proc Natl Acad Sci U S A* **115**, 1376-1381 (2018).
